# Supplementary material for: Bacterial Infections, Trends, and Resistance Patterns in the Time of the COVID-19 Pandemic in Romania—A Systematic Review
Source: Antibiotics (Basel). 2024 Dec 14;13(12):1219. doi: 10.3390/antibiotics13121219 (PMC11726834; doi:10.3390/antibiotics13121219)
Supplement: Supplementary file 1 [file antibiotics-13-01219-s001.zip › antibiotics-3302971-supplementary.pdf]

Table S1. Results of data on all selected articles and their demographics [~~16~~20-~~102~~106]

| No. | Type | Quality | Period | Patients (n) | Age | Sex | Location | Region |
|---|---|---|---|---|---|---|---|---|
| 1 [20] | CR | high | Jan 2021<br>pandemic | 1 | 23 | F | Galati | SE |
| 2 [21] | OA | fair | Jan 2018 –<br>Dec 2022<br>Pre-pandemic | Samples: 5548 | NR | NR | Bucharest | B |
| 3 [22] | CR | high | April 2022<br>pandemic | 1 | 42 | F | Dolj | SV |
| 4 [23] | OA | high | Jan - Dec 2022<br>pandemic | 111 | Mean: 72.1 | M: 50<br>F: 61 | Sibiu | C |
| 5 [24] | OA | fair | Jan 2014 –<br>Jan 2024<br>Pre-pandemic | 19 | Median: 8.1<br>IQR: 5.2-12.1 | M: 9<br>F: 10 | Timis | V |
| 6 [25] | OA | high | Jul - Dec 2021<br>pandemic | Total: 407<br>Bacterial confirmed: 113 | Mean bacterial: 64.5<br>+/- 12.9 | M: 62<br>F: 51 | Timis | V |
| 7 [26] | OA | fair | Nov 2020 –<br>Apr 2021<br>pandemic | 98 | NR | M: 52<br>F: 46 | Cluj | C |
| 8 [27] | OA | fair | 2002 - 2022<br>Pre-pandemic | 28 | 43.36 +/- 19.14 | M: 9<br>F: 19 | Iasi | NE |
| 9 [28] | OA | fair | Sep - Nov 2021<br>pandemic | Total: 280<br>Empirical ATB: 227<br>co-infections: 4<br>superinfections: 19 | 60.4 +/- 15.2 | M: 138<br>F: 142 | Iasi | NE |
| 10 [29] | OA | fair | Jan 2017 –<br>Dec 2022<br>Pre-pandemic | 152 | 60-80: 63%<br>40-60: 23.8%<br>80+: 6%<br>-40: 7.2% | M: 94<br>F: 49 | Bucharest | B |

| | | | | | | | | |
|---|---|---|---|---|---|---|---|---|
| 11 [30] | OA | fair | Jan 2018 – Dec 2021 Pre-pandemic | Total: 209 Bacterial: 181 | 63.6 +/- 9.5 | M: 110 F: 99 | Timis | V |
| 12 [31] | OA | high | Jan - May 2021 pandemic | Total: 553 Bacterial confirmed: 95 | Median: 67, min/max: 18-94 | M: 283 F: 270 | Multicentric | |
| 13 [32] | OA | fair | May 2021 – Aug 2023 Post-pandemic | Total: 148 Dual positivity: 69 | 45.93 +/- 12.83 | M: 61 F: 87 | Multicentric | |
| 14 [33] | OA | high | 2016 - 2020 Pre-pandemic | n= 91 Episodes: 260 | Median: 16 mo, min/max 10 days - 17.1 years | M:46 F: 45 | Timis | V |
| 15 [34] | OA | fair | Oct - Dec 2020 Oct - Dec 2021 pandemic | Total: 184 Co-infections: 18 | Median: 68, min/max: 34-93 | M: 98 F: 86 | Iasi | NE |
| 16 [35] | OA | fair | Nov 2021 – Jan 2022 Pre-pandemic | Total: 205 Empirical ATB: 84 Bacterial confirmed: 22 | Median: 66, IQR: 53-73 | M: 97 F: 108 | Bucharest | B |
| 17 [36] | OA | high | Jan 2017 – Dec 2022 pandemic | Samples: 1672 | NR | NR | Bucharest | B |
| 18 [37] | OA | high | May - Dec 2021 pandemic | 152 | 30.9 +/- 7 | F: 152 | Bucharest | B |
| 19 [38] | OA | low | 2011 - 2020 Pre-pandemic | 516 | Median: 57, IQR: 35 - 70 | M: 310 F: 206 | Bucharest | B |

| | | | | | | | | |
|---|---|---|---|---|---|---|---|---|
| 20 [39] | OA | fair | Jan 2016 – Oct 2022 Pre-pandemic | n= 67 Bacterial confirmed samples: 68 | 68.5 +/- 10.88 | M: 34 F: 33 | Sibiu | C |
| 21 [40] | OA | fair | Oct - Nov 2021 pandemic | 61 | 62.39 +/- 15.61 | M: 33 F: 28 | Timis | V |
| 22 [41] | OA | high | Jan 2022 – Dec 2023 Post-pandemic | n= 223 Samples: 226 | 18-30: 31 30-45: 24 46-60: 20 >60: 148 | M: 108 F: 115 | Dolj | SV |
| 23 [42] | OA | high | Oct 2020 – May 2021 pandemic | Total: 236 Superinfection: 119 | Median: 66.50, IQR: 58-72 | M: 58.90% F: 41.10% | Timis | V |
| 24 [43] | CR | fair | May 2021 pandemic | 1 | 73 | F | Constanta | SE |
| 25 [44] | OA | fair | Jan 2022 – Dec 2022 pandemic | Samples: 1445 Bacterial confirmed: 1435 | children | NR | Bucharest | B |
| 26 [45] | OA | fair | Jan 2016 – Dec 2022 Pre-pandemic | n= 60 Bacterial confirmed: 59 | >65: 39% | M: 26 F: 34 | Timis | V |
| 27 [46] | OA | fair | Sep 2022 – Feb 2023 Post-pandemic | 66 | 60.45 +/- 11.5 | M: 35 F: 31 | Timis | V |
| 28 [47] | CS | high | NR pandemic | 9 | 65 +/- 9.47 | M: 4 F: 5 | Constanta | SE |
| 29 [48] | OA | fair | Jan 2022 – Oct 2023 Post-pandemic | 162 | 67.4 +/- 15.37 | M: 94 F: 68 | Bucharest | B |
| 30 [49] | OA | fair | Mar - Dec 2020 pandemic | Episodes: 782 | NR | NR | Bucharest | B |

| | | | | | | | | |
|---|---|---|---|---|---|---|---|---|
| 31 [50] | OA | fair | Jan 2010 – Dec 2022 Pre-pandemic | Total: 1692 Bacterial confirmed: 307 | 0-20: 10 20-30: 37 30-40: 53 40-50: 56 50-60: 46 >60: 12 | M: 118 F: 96 | Iasi Bacau Botosani Suceava Neamt Vaslui | NE |
| 32 [51] | OA | fair | Jan 2019 – Dec 2020 Pre-pandemic | Total: 564 Bacterial confirmed: 73 | 68.63 +/- 17.2 | M: 179 F: 384 | Bucharest | B |
| 33 [52] | OA | fair | Sep 2020 – Apr 2021 pandemic | 109 | 57.72 +/- 17.33 | M: 44 F: 65 | Timis | V |
| 34 [53] | OA | fair | Sep 2019 – Sep 2022 Pre-pandemic | 140 | 64.42 +/-16.31 | M: 74 F: 66 | Iasi | NE |
| 35 [54] | OA | fair | Mar - Jun 2020 pandemic | Total: 132 Co-infections: 32 | 59.9 +/- 12.82 | M: 73 F: 59 TB M: 21 TB F: 11 | Timis | V |
| 36 [55] | OA | high | Aug 2020 – Aug 2023 Post-pandemic | 488 | 69.3 +/-10.9 | M: 227 F: 261 | Timis | V |
| 37 [56] | OA | high | 2019 - 2023 Pre-/ Post-pandemic | 308 | 27.9 +/- 4.6 | F: 308 | Timis | V |
| 38 [57] | OA | high | 2017 - 2022 Pre-pandemic | 34 | 45.37 +/- 7.73 | M: 21 F: 13 | Timis | V |
| 39 [58] | OA | fair | 2021 - 2022 pandemic | 212 | Mean: 59 Median 62 | M: 140 F: 72 | Bucharest | B |
| 40 [59] | OA | fair | Oct 2020 – Jun 2022 pandemic | Total: 973 Bacterial confirmed: 702 | Mean: 47.76 min/max: 12, 95 | M: 747 F: 226 | Brasov | C |
| 41 [60] | OA | fair | Apr 2020 – March 2022 pandemic | 3002 | 68.81 +/- 14.54 | NR | Mures | C |

| | | | | | | | | |
|---|---|---|---|---|---|---|---|---|
| 42 [61] | OA | low | Apr 2020 – Jun 2021 pandemic | n= 82 Samples: 93 | 65.51 +/- 11.23 | M: 49 F: 33 | Arad | W |
| 43 [62] | OA | fair | Jan - Jul 2021 pandemic | 79 | NR | NR | Cluj | C |
| 44 [63] | OA | high | Mar - Dec 2020 pandemic | 447 | 59.7 +/- 10.8 | M: 243 F: 204 | Iasi | NE |
| 45 [64] | OA | fair | Sep 2018 – Feb 2019 Sep 2020 – Feb 2021 Pre-pandemic | Pre-pandemic samples: 1505 | NR | F: 2469 | Bucharest | B |
| 46 [65] | OA | fair | Jan 2015 – Dec 2021 Pre-pandemic | 1082 | 12-15: 279 16-18: 803 | F: 1082 | Iasi | NE |
| 47 [66] | OA | fair | Jan 2017 – Jul 2022 Pre-pandemic | Total samples: 1994 | 66.16 +/-16.77 | M: 1073 F: 921 | Mures | C |
| 48 [67] | OA | fair | Oct 2018 – May 2022 Pre-pandemic | Total: 202 Bacterial confirmed: 200 Samples: 645 | 58 | M: 132 F: 70 | Bucharest | B |
| 49 [68] | OA | fair | 2017 - 2021 Pre-pandemic | Total: 4603 Bacterial confirmed: 2301 | 60.27 +/- 21.67 | M: 2454 F: 2149 | Mures | C |
| 50 [69] | OA | fair | Jan 2019 – Apr 2020 Pre-pandemic | 90 | 48.49 +/- 11.59 | NR | Dolj | SV |
| 51 [70] | OA | fair | Jan 2019 – Dec 2021 Pre-pandemic | 706 | 57.70 +/- 19.70 | M: 330 F: 376 | Bucharest | B |
| 52 [71] | OA | fair | Sep 2019 – May 2022 Pre-pandemic | 102 | 60.78 +/- 15.99 | M: 37 F: 33 | Galati | SE |

| | | | | | | | | |
|---|---|---|---|---|---|---|---|---|
| 53 [72] | OA | high | Jan 2020 – Mar 2022 Pre-pandemic | 86 | 67.61 | M: 43 F: 43 | Sibiu | C |
| 54 [73] | OA | fair | Mar 2017 – Feb 2018 Mar 2020 – Feb 2021 Pre-pandemic | Pre-pandemic: 99 | Prepandemic: median -62, IQR: 39-76 pandemic: median -66, IQR: 60-72 | M: 75 F: 75 | Bucharest | B |
| 55 [74] | OA | fair | Feb 2019 – Jun 2020 Pre-pandemic | 185 | 13.12 +/- 3.27 | M: 59 F: 126 | Iasi | NE |
| 56 [75] | OA | fair | 2017 - 2022 Pre-pandemic | 121 | 62.98 | M: 48 F: 73 | Bihor | NW |
| 57 [76] | OA | fair | Jun 2018 – Jun 2020 Pre-pandemic | 262 | Median: 70, IQR: 19 | M: 128 F: 134 | Timis | V |
| 58 [77] | OA | low | Mar - Nov 2018 Mar - Nov 2019 Mar - Nov 2020 Pre-pandemic | Pre-pandemic: 151 | NR | NR | Timis | V |
| 59 [78] | OA | fair | Mar 2020 – Nov 2022 pandemic | Samples: 116 Bacterial confirmed: 108 | NR | NR | Bucharest | B |
| 60 [79] | OA | fair | 2005 - 2022 Pre-pandemic | 107 | 57.7 | M: 58 F: 39 | Timis | V |
| 61 [80] | CR | high | 2021 pandemic | 1 | 45 | M | Timis | V |
| 62 [81] | OA | high | Mar 2020 – Aug 2022 pandemic | 88 | 63.64 +/- 12.57 | M: 47 F: 41 | Iasi | NE |

| | | | | | | | | |
|---|---|---|---|---|---|---|---|---|
| 63 [82] | OA | fair | 1990 - 2021<br>Pre-pandemic | 1990-2009: 199,<br>Bacterial confirmed: 175<br>2010-2021: 103,<br>Bacterial confirmed: 95 | neonates: 302 | M: 160<br>F: 142 | Iasi | NE |
| 64 [83] | OA | fair | Apr 2020 –<br>Dec 2020<br>pandemic | 80 | 65.91 +/- 14.31 | M: 44<br>F: 36 | Sibiu | C |
| 65 [84] | CR | fair | Jul 2021<br>pandemic | 1 | 73 | M | Galati | SE |
| 66 [85] | OA | high | Apr 2019 –<br>Mar 2020<br>Jul 2021 –<br>Jun 2022<br>Pre-pandemic | Pre-pandemic n= 1267<br>Samples: 1940 | 64 +/- 18.06 | M: 1638<br>F: 983 | Dolj | SV |
| 67 [86] | OA | fair | 2016 - 2020<br>Pre-pandemic | Samples: 4293 | NR | NR | Galati | SE |
| 68 [87] | OA | fair | Jan 2017 –<br>Dec 2020<br>Pre-pandemic | n= 491<br>Samples: 617 | 59 +/- 18.46 | M: 279<br>F: 212 | Dolj | SV |
| 69 [88] | OA | fair | Jan - Jun 2020<br>pandemic | 50 | 61.04 +/- 20.09 | M: 25<br>F: 25 | Iasi | NE |
| 70 [89] | OA | fair | Sep 2017 –<br>Oct 2021<br>Pre-pandemic | Pre-pandemic: 24 | 64.28 | M: 45<br>F: 19 | Constanta | SE |
| 71 [90] | OA | fair | Oct 2019 –<br>Oct 2020<br>Pre-pandemic | Samples: 72 | NR | NR | Bucharest | B |
| 72 [91] | OA | fair | Jan 2022 –<br>Aug 2023<br>Post-pandemic | 161 | NR | NR | Bucharest | B |

| | | | | | | | | |
|---|---|---|---|---|---|---|---|---|
| 73 [92] | OA | fair | Mar 2020 – Jan 2021 pandemic | 86 | 69.07 | M: 45 F: 41 | Iasi | NE |
| 74 [93] | OA | fair | Jan 2020 – Dec 2021 pandemic | Samples: 46 | NR | NR | Dolj | SV |
| 75 [94] | OA | low | Jan 2016 – Jun 2022 Pre-pandemic | 40 | 65.72 +/- 12.21 | M: 40 | Iasi Neamt | NE |
| 76 [95] | OA | fair | Jan 2016 – Dec 2021 Pre-pandemic | 171 | 61.75 +/- 7.95 | M: 103 F: 68 | Timis | V |
| 77 [96] | OA | high | Jan 2021 – Jun 2021 pandemic | n= 657 Bacterial confirmed samples: 858 | 62.38 +/- 10 | M: 355 F: 302 | Timis | V |
| 78 [97] | OA | high | Jan 2019 – Jun 2022 Pre-pandemic | Pre-pandemic: 46 | 69.20 +/- 12.30 | M: 43 F: 44 | Iasi | NE |
| 79 [98] | OA | fair | 2019 - 2020 Pre-pandemic | 149 | 6-9 years: 26 10-14 years: 53 15-17 years: 70 | M: 46 F: 103 | Iasi | NE |
| 80 [99] | OA | fair | Mar - Dec 2020 pandemic | Total: 198 Empirical ATB: 75 Bacterial confirmed: 21 | median: 61, range: 23-91 | M: 109 F: 89 | Bucharest | B |
| 81 [100] | OA | fair | Jan 2019 – Feb 2020 Mar 2020 – May 2021 Pre-pandemic | Pre-pandemic: 100 | 67.20 +/- 14.09 | M: 93 F: 95 | Mures | C |
| 82 [101] | OA | fair | Jul 2020 – | 90 | 52.10 +/- 14.69 | M: 71 F: 19 | Galati | SE |

| | | | | | | | | |
|---|---|---|---|---|---|---|---|---|
| | | | Jun 2022<br>pandemic | | | | | |
| 83 [102] | OA | fair | Jan 2020 –<br>Jan 2022<br>pandemic | Total: 489<br>Co-infections: 210<br>Super-infections: 279 | 18-40: 77<br>40-65: 224<br>>65: 188 | M: 271<br>F: 218 | Timis | V |
| 84 [103] | CR | fair | Jul 2021<br>pandemic | 1 | 31.00 | NR | Olt<br>Dolj | SV |
| 85 [104] | CR | high | 2020 - 2021<br>pandemic | 3 | 51.66 +/- 24.01 | M: 3 | Constanta | SE |
| 86 [105] | OA | fair | Jan 2022 –<br>Sept 2023<br>Post-pandemic | 246 | <1 year: 148<br><18: 98 | M: 136<br>F: 110 | Brasov | C |
| 87 [106] | OA | fair | Sep - Dec 2018<br>Sep - Dec 2020<br>Sep - Dec 2022<br>Pre-pandemic | Pre-pandemic: 478 | 19-29: 67<br>30-45: 132<br>45-59: 235<br>60+: 690 | F: 1124 | Bucharest | B |

Table S2. Outcomes

| No. | Period | Hospitalization | ICU (n) | ICU days | Death (n) |
|---|---|---|---|---|---|
| 1 [20] | Jan 2021<br>pandemic | 6 weeks | NR | | 0 |
| 2 [21] | Jan 2018 - Dec 2022<br>Pre-pandemic | NR | NR | | |
| 3 [22] | April 2022<br>pandemic | 20 days (total) | NR | | 0 |
| 4 [23] | Jan - Dec 2022<br>pandemic | 20.18 days | 4 | 3.45 | 28 |
| 5 [24] | Jan 2014 - Jan 2024<br>Pre-pandemic | Median: 9, IQR: 5.5-21 | NR | | 0 |
| 6 [25] | Jul - Dec 2021<br>pandemic | 11.4 +/- 3.3 | 36 | 7.6 +/- 2 | 28 |

| | | | | | |
|---|---|---|---|---|---|
| 7 [26] | Nov 2020 - Apr 2021 pandemic | Mean: 31.85 | NR | NR | 50 |
| 8 [27] | 2002 - 2022 Pre-pandemic | NR | NR | NR | |
| 9 [28] | Sep - Nov 2021 pandemic | Median: 10, IQR: 10-11 | total: 41 Bacterial infections: 19 | | Total: 31 Bacterial infections: 6 |
| 10 [29] | Jan 2017 - Dec 2022 Pre-pandemic | NR | NR | NR | NR |
| 11 [30] | Jan 2018 - Dec 2021 Pre-pandemic | 16.6 +/- 5.7 | 109 | 6.8 +/- 3.1 | 74 |
| 12 [31] | Jan - May 2021 pandemic | Median: 12.5, IQR: 2.5-49 | NR | NR | Total: 48 ATB: 39 |
| 13 [32] | May 2021 - Aug 2023 Post-pandemic | NR | NR | NR | NR |
| 14 [33] | 2016 - 2020 Pre-pandemic | NR | NR | NR | NR |
| 15 [34] | Oct - Dec 2020 Oct - Dec 2021 pandemic | Median: 5 days (1-10) | 140 | Median: 9 days (3-20) | 127 |

| | | | | | |
|---|---|---|---|---|---|
| 16 [35] | Nov 2021 - Jan 2022 Pre-pandemic | NR | 18 | NR | NR |
| 17 [36] | Jan 2017 - Dec 2022 pandemic | NR | NR | NR | NR |
| 18 [37] | May - Dec 2021 pandemic | NR | NR | NR | NR |
| 19 [38] | 2011 - 2020 Pre-pandemic | Median: 10 days, 3 - 10 | 401 | Median: 10 days, 2 - 10 | 516 |
| 20 [39] | Jan 2016 - Oct 2022 Pre-pandemic | NR | NR | NR | NR |
| 21 [40] | Oct - Nov 2021 pandemic | NR | NR | NR | NR |
| 22 [41] | Jan 2022 - Dec 2023 Post-pandemic | NR | NR | NR | NR |
| 23 [42] | Oct 2020 - May 2021 pandemic | NA | 119 | Median: 9, IQR: 6-12.50 | Total: 179 bacterial superinfections: 88 |
| 24 [43] | May 2021 pandemic | 25 days | NR | NR | 1 |

| | | | | | | |
|---|---|---|---|---|---|---|
| 25 [44] | Jan 2022 - Dec 2022<br>pandemic | NR | 379 | NR | NR | |
| 26 [45] | Jan 2016 - Dec 2022<br>Pre-pandemic | NR | NR | NR | NR | |
| 27 [46] | Sep 2022 - Feb 2023<br>Post-pandemic | NR | NR | NR | NR | |
| 28 [47] | NR<br>pandemic | Median: 23.22 days, IQR: 10-48 | 9 | Median: 16.4<br>days, IQR: 4-46 | | 5 |
| 29 [48] | Jan 2022 - Oct 2023<br>Post-pandemic | 25.80 +/- 18.77 | 35 | NR | | 50 |
| 30 [49] | Mar - Dec 2020<br>pandemic | Total: 88767<br>14.35 blood culture/ 1000 patient-days/ CD | 220 | NR | NR | |
| 31 [50] | Jan 2010 - Dec 2022<br>Pre-pandemic | Median: 18 days | NA | NA | | 97 |
| 32 [51] | Jan 2019 - Dec 2020<br>Pre-pandemic | 8.33 days +/- 5.82 | NR | NR | | 42 |
| 33 [52] | Sep 2020 - Apr 2021<br>pandemic | 32, range: 1-58 | NR | NR | total: 30<br>C difficle: 9 | |
| 34 [53] | Sep 2019 - Sep 2022<br>Pre-pandemic | NR | 4 | 10 days | | 20 |

| | | | | | |
|---|---|---|---|---|---|
| 35 [54] | Mar - Jun 2020<br>pandemic | NR | NR | NR | 25<br>TB: 8 |
| 36 [55] | Aug 2020 - Aug 2023<br>Post-pandemic | median: 8.6 days IQ: 5.3 | NR | NR | NR |
| 37 [56] | 2019 - 2023<br>Pre-/ Post-pandemic | NR | NR | NR | NR |
| 38 [57] | 2017 - 2022<br>Pre-pandemic | NA | NA | NA | NA |
| 39 [58] | 2021 - 2022<br>pandemic | 17.5 days<br>non-MDR: 10<br>MDR: 25 | NA | NA | NR |
| 40 [59] | Oct 2020 - Jun 2022<br>pandemic | Mean: 10, min/max: 1, 101 | NR | NR | NR |
| 41 [60] | Apr 2020 - March 2022<br>pandemic | NA | 3002<br>C. difficle: 100 | 11.39 +/- 8.68 | 36 |
| 42 [61] | Apr 2020 - Jun 2021<br>pandemic | Median: 11 days, IQR: 6-17 | 66 | NR | NR |
| 43 [62] | Jan - Jul 2021<br>pandemic | NR | NR | NR | NR |
| 44 [63] | Mar - Dec 2020<br>pandemic | 8.5 +/- 6 | NR | NR | NR |
| 45 [64] | Sep 2018 - Feb 2019<br>Sep 2020<br>Feb 2021<br>Pre-pandemic | NR | NR | NR | NR |
| 46 [65] | Jan 2015 - Dec 2021<br>Pre-pandemic | NR | NR | NR | NR |

| | | | | | |
|---|---|---|---|---|---|
| 47 [66] | Jan 2017 - Jul 2022<br>Pre-pandemic | NR | 239<br>2020: 11<br>2021: 53<br>2022: 23 | NR | NR |
| 48 [67] | Oct 2018 - May 2022<br>Pre-pandemic | NR | 202 | NR | 133 |
| 49 [68] | 2017 - 2021<br>Pre-pandemic | NR | 1486 | NR | 1176 |
| 50 [69] | Jan 2019 - Apr 2020<br>Pre-pandemic | NR | NA | NA | NR |
| 51 [70] | Jan 2019 - Dec 2021<br>Pre-pandemic | NR | NR | NR | NR |
| 52 [71] | Sep 2019 - May 2022<br>Pre-pandemic | NR | 64 | NR | 14 |
| 53 [72] | Jan 2020 - Mar 2022<br>Pre-pandemic | NR | NR, excluded | NR | NR, excluded |
| 54 [73] | Mar 2017 - Feb 2018<br>Mar 2020 - Feb 2021<br>Pre-pandemic | prepandemic: median -14, IQR: 10-24<br>pandemic: median -19, IQR: 15-24 | NR | NR | prepandemic: 4<br>pandemic: 0 |
| 55 [74] | Feb 2019 - Ju 2020<br>Pre-pandemic | 14 days | NA | NA | NR |
| 56 [75] | 2017 - 2022<br>Pre-pandemic | NR | NA | NA | NR |
| 57 [76] | Jun 2018 - Jun 2020<br>Pre-pandemic | Median: 7, IQR: 4-10 | NR | NR | NR |

| | | | | | |
|---|---|---|---|---|---|
| 58 [77] | Mar - Nov 2018<br>Mar - Nov 2019<br>Mar - Nov 2020<br>Pre-pandemic | NR | NR | NR | NR |
| 59 [78] | Mar 2020 - Nov 2022<br>pandemic | NR | NR | NR | NR |
| 60 [79] | 2005 - 2022<br>Pre-pandemic | NR | NR | NR | NR |
| 61 [80] | 2021<br>pandemic | 21 | x | NA | x |
| 62 [81] | Mar 2020 - Aug 2022<br>pandemic | 7.78 +/- 4.9 | NR | NR | NR |
| 63 [82] | 1990 - 2021<br>Pre-pandemic | Median: 13.5, IQR: 5-26 | 302 | Median: 13.5, IQR: 5-26 | CD |
| 64 [83] | Apr 2020 - Dec 2020<br>pandemic | 19.63 +/- 9.06 | NR | NR | 20 |
| 65 [84] | Jul 2021<br>pandemic | 19 | NR | NR | x |
| 66 [85] | Apr 2019 - Mar 2020<br>Jul 2021 - Jun 2022<br>Pre-pandemic | NA | 2621 patients | NR | NR |
| 67 [86] | 2016 - 2020<br>Pre-pandemic | NR | NR | NR | NR |

| | | | | | |
|---|---|---|---|---|---|
| 68 [87] | Jan 2017 - Dec 2020 Pre-pandemic | NR | 491 patients 617 samples | NR | NR |
| 69 [88] | Jan - Jun 2020 pandemic | 12.37 +/- 8.69 | NR | NR | 7 |
| 70 [89] | Sep 2017 - Oct 2021 Pre-pandemic | NA | 64 | Median: 9.54 days | 34 |
| 71 [90] | Oct 2019 - Oct 2020 Pre-pandemic | NR | NR | NR | NR |
| 72 [91] | Jan 2022 - Aug 2023 Post-pandemic | NR | NA | NA | NR |
| 73 [92] | Mar 2020 - Jan 2021 pandemic | 14.44 | 13 | NR | 9 |
| 74 [93] | Jan 2020 - Dec 2021 pandemic | NR | 46 | NR | NR |
| 75 [94] | Jan 2016 - Jun 2022 Pre-pandemic | 18.25 +/- 9.03 | NR | NR | 5 |
| 76 [95] | Jan 2016 -Dec 2021 Pre-pandemic | 17 +/- 5.65 | 82 | 6.78 +/- 2.81 | 66 |
| 77 [96] | Jan 2021 - Jun 2021 pandemic | NR | 77 | NR | NR |
| 78 [97] | Jan 2019 - Jun 2022 Pre-pandemic | 20 +/- 17.70 days | 26 | NR | 15 |
| 79 [98] | 2019 - 2020 Pre-pandemic | NR | NR | NR | NR |

| | | | | | | |
|---|---|---|---|---|---|---|
| 80 [99] | Mar - Dec 2020<br>pandemic | NR | NR | NR | NR | |
| 81 [100] | Jan 2019 - Feb 2020<br>Mar 2020 - May 2021<br>Pre-pandemic | Median: 11.5, range: 1-50 | 42 | NR | | 44 |
| 82 [101] | Jul 2020 - Jun 2022<br>pandemic | NR | NR | NR | NR | |
| 83 [102] | Jan 2020 - Jan 2022<br>pandemic | 12.4 +/- 4.85 | 33 | 12.25 +/- 5.85 | | 27 |
| 84 [103] | Jul 2021<br>pandemic | NR | NR | NR | NR | |
| 85 [104] | 2020 - 2021<br>pandemic | 15.67 +/- 10.07 | x | x | x | |
| 86 [105] | Jan 2022 - Sept 2023<br>Post-pandemic | 6.50 +/- 3.53 | 2/ 14 | NR | | 4 |
| 87 [106] | Sep - Dec 2018<br>Sep - Dec 2020<br>Sep - Dec 2022<br>Pre-pandemic | NR | NR | NR | NR | |

Table S3. Origins of bacterial strains

| No. | Period | Ward/ department | Infection location | ID method | Bacteria |
|---|---|---|---|---|---|
| 1 [20] | Jan 2021<br>pandemic | Cardiology | BSI | Culture | S. gordonii |
| 2 [21] | Jan 2018 –<br>Dec 2022<br>Pre-pandemic | NR | UTI | Vitek 2 compact<br>MALDI TOF MS | E. coli: 56.43%<br>K. pneumoniae: 16.64%<br>Other Klebsiella spp.: 1.06% |

| | | | | | |
|---|---|---|---|---|---|
| | | | | | E. faecalis: 7.69%<br>E. faecium: 2.38%<br>P. aeruginosa: 4.59%<br>A. baumanii: 0.68%<br>Enterobacter spp., Proteus spp., Morganella spp., S. saprophyticus, other: 10.53% |
| 3 [22] | April 2022<br>pandemic | Ophthalmology | Ocular | Vitek 2 compact | R. radiobacter |
| 4 [23] | Jan - Dec 2022<br>pandemic | Int. Med.: 30<br>Surgery: 26<br>Inf. Dis.: 22<br>Neurology: 7<br>Others: 26 | Surgical: 38<br>Non-surgical: 73 | NR | C. difficile |
| 5 [24] | Jan 2014 –<br>Jan 2024<br>Pre-pandemic | Pediatrics | NA | Serology | B. henselae |
| 6 [25] | Jul - Dec 2021<br>pandemic | Inf. Dis. | Upper Respiratory<br>Sputum | Vitek 2 compact | P. aeruginosa: 12.8%<br>K. pneumoniae: 10.7%<br>E. coli: 6%<br>A. baumanii: 3.8%<br>Other: 8.4%<br>S. pneumoniae: 20.2%<br>S. aureus: 22.9%<br>Other Streptococcus spp.: 5.5%<br>M. catarrhalis: 5.4%<br>H. influenzae: 3.9%<br>Other commensal: 1.5% |
| 7 [26] | Nov 2020 –<br>Apr 2021<br>pandemic | NR | Blood: 37<br>Wounds: 35<br>UTI:4<br>CSF: 4<br>CVC: 4<br>Puss: 3<br>Puncture: 1<br>Peritoneal: 6<br>Pleural: 1<br>BAL/ TA: 3 | Vitek 2 compact | E. faecalis: 54<br>E. faecium: 42<br>E. gallinarum: 2 |
| 8 [27] | 2002 - 2022<br>Pre-pandemic | Surgery | Digestive tract | Pathology<br>Culture | A. israelii |

| | | | | | | |
|---|---|---|---|---|---|---|
| 9 [28] | Sep - Nov pandemic | 2021 | NR | BSI: 3<br>Respiratory: 12<br>UTI: 12 | Culture | A. baumanii: 6<br>K. pneumoniae: 5<br>S. aureus: 1<br>P. aeruginosa: 5<br>E. coli: 3<br>E. faecalis: 3 |
| 10 [29] | Jan 2017 – Dec Pre-pandemic | 2022 | NR | BSI | Serology: 9<br>Culture: 143 | C. burnetti: 9<br>E. faecalis: 38<br>Other Enterococcus spp.: 5<br>E. coli: 7<br>K. pneumoniae: 1<br>P. aeruginosa: 1<br>S. aureus: 32<br>CoNS: 14<br>Group B strep.: 1<br>Group C strep.: 1<br>Group D strep.: 6<br>Viridans strep.: 36 |
| 11 [30] | Jan 2018 – Dec Pre-pandemic | 2021 | NR | BSI | Culture<br>PCR | S. aureus: 60<br>CoNS: 26<br>Streptococcus spp.: 40<br>E. coli: 12<br>E. faecalis: 29<br>Other GNB: 14 |
| 12 [31] | Jan - May pandemic | 2021 | NR | Respiratory, sputum: 38<br>UTI: 31<br>Blood: 39 | Culture | S. aureus: 11<br>CoNS: 8<br>S. pneumoniae: 5<br>Other Streptococcus spp.: 5<br>Enterococcus spp.: 4<br>K. pneumoniae: 26<br>E. coli: 25<br>P. aeruginosa: 4<br>A. baumanii: 3<br>H. influenzae: 4<br>S. maltophilia: 3<br>Serratia spp.: 2<br>K. oxytoca: 1<br>Other GNB: 7<br>C. difficile: 48 |
| 13 [32] | May 2021 – | | GE | Gastric biopsy | PCR<br>Culture | H. pylori |

| | | | | | |
|---|---|---|---|---|---|
| | Aug 2023<br>Post-pandemic | | | | |
| 14 [33] | 2016 - 2020<br>Pre-pandemic | Pediatrics | UTI | Culture | Total:<br>E. coli: 101<br>Klebsiella spp.: 55<br>Enterobacter spp.: 6<br>Proteus spp.: 21<br>Serratia spp.: 3<br>Citrobacter spp.: 2<br>Morganella spp.: 1<br>Pseudomonas spp.: 21<br>S. maltophilia: 2<br>R. pickettii: 1<br>Enterococcus spp.: 41<br>S. aureus: 4<br>Group A strep.: 2<br><br>2020:<br>E. coli: 8<br>Klebsiella spp.: 4<br>Proteus spp.: 10<br>Pseudomonas spp.: 4<br>Enterococcus spp.: 4<br>Gorup A strep.: 1 |
| 15 [34] | Oct - Dec 2020<br>Oct - Dec 2021<br>pandemic | Inf. Dis. | Pulmonary: 60<br>UTI: 15<br>Skin: 2<br>BSI: 5 | NR | P. aeruginosa: 5<br>A. baumanii: 3<br>K. pneumoniae: 4<br>S. aureus: 2<br>E. coli: 2<br>Proteus spp.: 2<br>C. difficile: 10 |
| 16 [35] | Nov 2021 –<br>Jan 2022<br>Pre-pandemic | Inf. Dis. | UTI: 15<br>Sputum: 6<br>BSI: 3 | Culture | E. coli: 10<br>E. faecalis: 5<br>P. stuartii: 1<br>P aeruginosa: 2<br>S. aureus: 4<br>H. influenzae: 1<br>Moraxella spp.: 1<br>Group G strep.: 1<br>K pneumoniae: 1<br>K. oxytoca: 1 |

| | | | | | | |
|---|---|---|---|---|---|---|
| | | | | | | E. cloacae: 1<br>A. baumanii: 1 |
| 17 [36] | Jan 2017 – Dec<br>pandemic | 2022 | Inf. Dis. | (total/2020/ 2021/2022)<br>Wounds: 966/ 54/ 25/ 129<br>Ocular: 134/ 6/ 4/ 15,<br>Sputum, bronchial aspirate, bronchoalveolar lavage: 157/ 15/ 43/ 33<br>Blood: 313/ 27/ 17/ 129<br>Pleural, joint fluids, ear secretions, UTI: 102/ 8/ 10/10 | BacT/ Alert<br>MALDI TOF MS | S. aureus: 1672 |
| 18 [37] | May - Dec<br>pandemic | 2021 | Ob-Gyn | Vaginal | Culture<br>Hemolysis<br>Group agglutination | Group B strep.: 152 |
| 19 [38] | 2011 -<br>Pre-pandemic | 2020 | Legal medicine | BSI<br>Respiratory<br>Skin<br>Wound | Culture<br>Histology | E faecium: 73<br>S. aureus: 54<br>K. pneumoniae: 154<br>A. baumanii: 107<br>P. aeruginosa: 126<br>Enterobacter spp.: 262 |
| 20 [39] | Jan 2016 – Oct<br>Pre-pandemic | 2022 | Orthopedics | Joint implants | Vitek 2 compact | CoNS: 19<br>S. aureus: 21<br>Group D strep.: 2<br>E. faecalis: 6<br>E. coli: 6<br>Enterobacter spp.: 8<br>Klebsiella spp.: 2<br>P. mirabilis: 3<br>Pseudomonas spp.: 9<br>A. baumanii: 3<br>R. picketti: 4 |
| 21 [40] | Oct - Nov<br>pandemic | 2021 | ENT<br>Inf. Dis. | Oral swab | Vitek 2 compact | S. aureus: 5<br>Group A strep.: 3<br>Group G strep: 6<br>Group C strep.: 8<br>H. influenzae: 1 |
| 22 [41] | Jan 2022 – Dec<br>Post-pandemic | 2023 | Ophthalmology | Corneal swab | Vitek 2 compact | CoNS: 80<br>S. aureus: 25<br>S. pneumoniae: 16 |

| | | | | | | |
|---|---|---|---|---|---|---|
| | | | | | | Viridans strep.: 8<br>P. aeruginosa: 32<br>K. pneumoniae: 18<br>E. coli: 6<br>P. mirabilis: 6<br>S. maltophilia: 4<br>S. marcescens: 4<br>C. freundii: 3<br>H. influenzae: 3<br>A. baumanii: 2<br>B. cepacia: 2<br>R. radiobacter: 1 |
| 23 [42] | Oct 2020 –<br>May<br>pandemic | 2021 | COVID-19 ICU | BAL/ TA: 65<br>BSI: 48<br>UTI: 24<br>Sputum: 9<br>Wounds: 4<br>Stool: 26<br>Other: 7 | Vitek 2 compact | A. baumanii: 35<br>K. pneumoniae: 13<br>Other Klebsiella spp.: 6<br>E. faecalis: 10<br>E. faecium: 5<br>Other enterococcus spp.: 12<br>P. aeruginosa: 10<br>Enterobacter spp.: 4<br>S. aureus: 9<br>CoNS: 31<br>C. difficile: 26 |
| 24 [43] | May<br>pandemic | 2021 | Inf. Dis. | BSI | NR | E. faecium |
| 25 [44] | Jan 2022 –<br>Dec<br>pandemic | 2022 | Cardiology<br>Oncology<br>Orthopedics<br>ENT<br>Pediatrics<br>Nephrology<br>Hemodialysis<br>Neurosurgery<br>ICU | UTI: 331<br>Respiratory: 262<br>Skin, nose, pharynx: 249<br>BSI: 182<br>Pus: 114<br>CVC: 87<br>Peritoneal fluid: 86<br>Stool: 48<br>Vaginal/ urethral: 22<br>CSF: 12<br>Others: 52 | Biochemical kits<br>MicroScan | E. coli: 343<br>S. aureus: 226<br>Klebsiella spp.: 174<br>Pseudomonas spp.: 144<br>CoNS: 128<br>Enterococcus spp.: 107<br>A. baumanii: 51<br>Proteus spp.: 42<br>Enterobacter spp.: 41<br>S. pneumoniae: 15<br>Salmonella spp.: 14<br>Other strep.: 27<br>S. marcescens: 10<br>H. influenzae: 2 |
| 26 [45] | Jan 2016 – | | Phlebology | Wounds | NR | P. aeruginosa: 34<br>P. mirabilis: 3 |

| | | | | | | |
|---|---|---|---|---|---|---|
| | Dec<br>Pre-pandemic | 2022 | | | | Enterococcus spp.: 3<br>S. aureus: 6<br>A. baumanii: 2<br>Klebsiella spp.: 3<br>E. coli: 4<br>P. rettgeri: 1<br>Group B strep.: 1<br>Salmonella spp.: 1<br>Morganella spp.: 1 |
| 27 [46] | Sep 2022 –<br>Feb<br>Post-pandemic | 2023 | Inf. Dis. | BAL | Multiplex PCR<br>Culture | Commensal flora: 54<br>P. aeruginosa: 3<br>Streptococcus spp.: 6<br>Acinetobacter spp.: 5<br>Klebsiella spp.: 11<br>S. aureus: 3<br>E. coli: 13<br>Serratia spp.: 2 |
| 28 [47] | NR<br>pandemic | | ICU | Sputum: 7<br>BSI: 1<br>UTI: 1 | Vitek 2 compact<br>MALDI TOF MS | K. pneumoniae |
| 29 [48] | Jan 2022 –<br>Oct<br>Post-pandemic | 2023 | Surgical: 40<br>Non-surgical: 91 | UTI: 95<br>Others: 67 | Culture<br>Vitek 2 compact<br>MALDI TOF MS | K. pneumoniae: 162<br>Pseudomonas spp.: 16<br>E. coli: 12<br>Acinetobacter spp.: 11<br>Proteus spp.: 6<br>Providencia spp.: 6 |
| 30 [49] | Mar - Dec<br>pandemic | 2020 | Inf. Dis.<br>ICU | BSI | NR | E. coli: 4<br>S. aureus: 9<br>K. pneumoniae: 16<br>Other Enterobacteriaceae: 3<br>Enterococcus spp.: 6<br>S. pneumoniae: 1<br>A. baumanii: 22<br>P. aeruginosa: 6<br>S. maltophilia: 2<br>Other: 10 |
| 31 [50] | Jan 2010 –<br>Dec<br>Pre-pandemic | 2022 | Inf. Dis.<br>HIV/ AIDS | CSF | PCR<br>RIF | TB: 214<br>T. pallidum: 15 |
| 32 [51] | Jan 2019 – | | Nephrology | UTI | NR | C. difficile: 34<br>E. coli: 385 |

| | | | | | |
|---|---|---|---|---|---|
| | Dec 2020 Pre-pandemic | | | | Pseudomonas spp.: 34 Staphylococcus spp.: 8 Streptococcus spp.: 4 Proteus spp.: 34 Klebsiella spp.: 63 Enterococcus spp.: 36 |
| 33 [52] | Sep 2020 – Apr 2021 pandemic | Inf. Dis. | Stool | VIDAS | C. difficile: 40 |
| 34 [53] | Sep 2019 – Sep 2022 Pre-pandemic | Surgery | Stool | ELFA | C. difficile: 140 |
| 35 [54] | Mar - Jun 2020 pandemic | Inf. Dis. | Respiratory | Culture | TB: 32 |
| 36 [55] | Aug 2020 – Aug 2023 Post-pandemic | GE | Bile | Vitek 2 | E. coli: 150 Klebsiella spp.: 90 Pseudomonas spp.: 33 Enterobacter spp.: 31 Acinetobacter spp.: 10 Citrobacter spp.: 17 Enterococcus spp.: 115 Streptococcus spp.: 19 Staphylococcus spp.: 20 |
| 37 [56] | 2019 - 2023 Pre-/ Post-pandemic | Ob-Gyn | UTI | Vitek 2 | E. coli: 190 Klebsiella spp.: 53 Pseudomonas spp.: 38 Enterobacter spp.: 45 Proteus spp.: 23 Bacteroides spp.: 18 Enterococcus spp.: 23 Group B strep.: 25 Other Streptococcus spp.: 22 Staphylococcus spp.: 11 |
| 38 [57] | 2017 - 2022 Pre-pandemic | Dentistry | Periodontal samples | micro-IDent PCR | A. actinomycetemcomitans: 7 P. gingivalis: 31 P. intermedia: 25 T. forsythia: 31 T. denticola: 32 |
| 39 [58] | 2021 - 2022 pandemic | Vascular surgery Plastic surgery Diabetes | Wounds | Culture | E. faecium: 7 S. aureus: 135 K. pneumoniae: 33 |

| | | | | | | |
|---|---|---|---|---|---|---|
| | | | Cardiology | | | A. baumanii: 8<br>P. aeruginosa: 51<br>Enterobacter spp.: 21 |
| 40 [59] | Oct 2020 – Jun pandemic | 2022 | Plastic surgery | Wounds | Vitek 2 | CoNS: 344<br>S. aureus: 191<br>Klebsiella spp.: 84<br>Enterococcus spp.: 81<br>P. aeruginosa: 58<br>E. coli: 42<br>Acinetobacter spp.: 38<br>Proteus spp.: 30<br>Streptococcus spp.: 23<br>Enterobacter spp.: 4<br>Serratia spp.: 3 |
| 41 [60] | Apr 2020 – March pandemic | 2022 | ICU | Stool | Immunologic | C. difficile: 368 |
| 42 [61] | Apr 2020 – Jun pandemic | 2021 | General | Sputum: 41<br>UTI: 8<br>BAL/ TA: 29<br>BSI: 9<br>Wounds: 6 | Vitek 2 compact | K. pneumoniae: 29<br>Acinetobacter spp.: 11<br>Enterococcus spp.: 18<br>E. coli: 8<br>Enterobacter spp.: 2<br>S. aureus: 11<br>S. marcescens: 2<br>CoNS: 9<br>P. aeruginosa: 5 |
| 43 [62] | Jan - Jul pandemic | 2021 | ICU | Rectal swab | Culture<br>MALDI TOF MS | E. faecium: 68<br>E. faecalis: 11 |
| 44 [63] | Mar - Dec pandemic | 2020 | Inf. Dis. | stool | immunologic | C. difficile: 447 |
| 45 [64] | Sep 2018 – Feb<br>Sep 2020 – Feb<br>Pre-pandemic | 2019<br>2021 | General | UTI | Culture<br>Biochemical | Prepandemic:<br>E. coli: 925<br>Klebsiella spp.: 243<br>Proteus spp.: 80<br>Pseudomonas spp.: 33<br>Enterococcus spp.: 191<br>Staphylococcus spp.: 33<br><br>Pandemic:<br>E. coli: 580<br>Klebsiella spp.: 183 |

| | | | | | |
|---|---|---|---|---|---|
| | | | | | Proteus spp.: 57<br>Pseudomonas spp.: 31<br>Enterococcus spp.: 94<br>Staphylococcus spp.: 19 |
| 46 [65] | Jan 2015 - Dec 2021<br>Pre-pandemic | Ob-Gyn | Vaginal: 481<br>UTI: 69 | NR | Citrobacter spp.: 3<br>E. coli: 230<br>G. vaginalis: 3<br>K. pneumoniae: 34<br>Enterococcus spp.: 23<br>Enterobacter spp.: 9<br>Group B strep.: 30<br>P. mirabilis: 2<br>P. aeruginosa: 1<br>S. aureus: 8<br>S. marcescens: 1 |
| 47 [66] | Jan 2017 –<br>Jul 2022<br>Pre-pandemic | General | Wounds: 1468<br>Low respiratory: 172<br>UTI: 260<br>BSI: 20<br>Others: 74 | Culture<br>Biochemical<br>Vitek 2 compact | Total:<br>P. aeruginosa: 1942<br>P. putida: 6<br>P. fluorescens: 9<br>Other Pseudomonas spp.: 39<br><br>2020<br>P. aeruginosa: 117<br><br>2021<br>P. aeruginosa: 293<br>P. putida: 3<br>P. fluorescens: 2<br>Other Pseudomonas spp.: 1<br><br>2022<br>P. aeruginosa: 130<br>P. putida: 2<br>P. fluorescens: 3<br>Other Pseudomonas spp.: 6 |
| 48 [67] | Oct 2018 –<br>May 2022<br>Pre-pandemic | ICU<br>Plastic surgery<br>Reconstructive surgery<br>Burns unit | Wounds: 353<br>TA: 184<br>UTI: 63<br>BSI: 46 | VersaTREK<br>REDOX<br>MicroScan<br>Walkaway 40 Plus | P. aeruginosa: 298<br>S. aureus: 88<br>Klebsiella spp.: 80<br>A. baumanii: 70<br>E. coli: 35 |

| | | | | | |
|---|---|---|---|---|---|
| | | | | | Proteus spp.: 34<br>Other GNB: 40 |
| 49 [68] | 2017 - 2021<br>Pre-pandemic | General | NR | NR | C. difficile: 1501<br>A. baumanii: 544<br>K. pneumoniae: 441<br>P. aeruginosa: 366<br>S. aureus: 247<br>E. coli: 153<br>Other GNB: 431 |
| 50 [69] | Jan 2019 – Apr 2020<br>Pre-pandemic | Pneumo-phtysiology | Sputum | Histology<br>Culture | TB: 90 |
| 51 [70] | Jan 2019 – Dec 2021<br>Pre-pandemic | Inf. Dis. | NR | MALDI TOF MS | K. pneumoniae: 706 |
| 52 [71] | Sep 2019 – May 2022<br>Pre-pandemic | General | UTI | Vitek<br>Culture | E. coli: 51<br>Klebsiella spp.: 27<br>Enterococcus spp.: 13<br>P. aeruginosa: 8<br>Proteus spp.: 2<br>Streptococcus spp.: 1 |
| 53 [72] | Jan 2020 – Mar 2022<br>Pre-pandemic | GE | Stool | Immunologic | C. difficile: 86 |
| 54 [73] | Mar 2017 – Feb 2018<br>Mar 2020 – Feb 2021<br>Pre-pandemic | Inf. Dis. | Stool | Immunologic<br>PCR | C. difficile:<br>Prepandemic: 99<br>Pandemic: 51 |
| 55 [74] | Feb 2019 – Ju 2020<br>Pre-pandemic | GE<br>Pediatrics | Gastric biopsy | Histopathology | H. pylori: 116 |
| 56 [75] | 2017 - 2022<br>Pre-pandemic | GE | Gastric biopsy | Biochemical- urease test<br>Histopathology | H. pylori: 105 |
| 57 [76] | Jun 2018 – Jun 2020<br>Pre-pandemic | GE | BSI: 141<br>bile: 262 | Vitek 2 | (bile + blood)<br>E. coli: 92 + 21<br>Klebsiella spp.: 51 + 9<br>Pseudomonas spp.: 25 + 2<br>Enterobacter spp.: 10 + 4<br>Acinetobacter spp.: 6 + 1<br>Citrobacter spp.: 11 + 1 |

| | | | | | | |
|---|---|---|---|---|---|---|
| | | | | | | Enterococcus spp.: 41 + 2<br>Streptococcus spp.: 6 + 1<br>Staphylococcus spp.: 3 + 4 |
| 58 [77] | Mar - Nov<br>Mar - Nov<br>Mar - Nov<br>Pre-pandemic | 2018<br>2019<br>2020 | General | Stool | NR | C. difficile:<br>2018-2019: 151<br>2020: 65 |
| 59 [78] | Mar 2020 – Nov<br>pandemic | 2022 | General | BSI: 116 | BacT/ Alert 3D<br>Multiplex PCR<br>Culture<br>MADLI TOF M | Micrococcus spp.: 1<br>Bacillus spp.: 8<br>S. maltophilia: 2<br>D. acidovorans: 1<br>A. aphophilus: 1<br>M. osloensis: 1<br>K. pnemoniae: 19<br>E. coli: 17<br>A. baumanii: 14<br>Enterococcus spp.: 12<br>S. aureus: 9 |
| 60 [79] | 2005 -<br>Pre-pandemic | 2022 | D-V | NA | IDR, Mantoux | TB: 30 |
| 61 [80] | 2021<br>pandemic | | Neurology<br>Inf. Dis. | CSF | MALDI TOF MS<br>Vitek 2 compact | L. monocytogenes: 1 |
| 62 [81] | Mar 2020 –<br>Aug<br>pandemic | 2022 | Urology | UTI | Culture | E. coli:35<br>Klebsiella spp.: 28<br>P. aeruginosa: 12<br>P. mirabilis: 4<br>Enterobacter spp.: 1<br>Providencia spp.: 1<br>S. marcescens: 1<br>Enterococcus spp.: 6 |
| 63 [82] | 1990 -<br>Pre-pandemic | 2021 | Neonatal ICU | Wounds | NR | Enterobacteriaceae: 20.2%<br>Pseudomonas spp.: 11.3%<br>Acinetobacter spp.: 11.3%<br>S. aureus: 8.3%<br>Enterococcus spp.: 2% |
| 64 [83] | Apr 2020 –<br>Dec<br>pandemic | 2020 | General | Stool | Immunologic | C. difficile: 80 |
| 65 [84] | Jul<br>pandemic | 2021 | Surgery | Wound | NR | E. coli |

| | | | | | |
|---|---|---|---|---|---|
| 66 [85] | Apr 2019 – Mar 2020<br>Jul 2021 – Jun 2022<br>Pre-pandemic | ICU | BSI: 767<br>UTI: 223<br>Respiratory: 2649<br>Puss/ Wounds: 339<br>Other: 243 | Bact/ Alert 3D<br>MADLI TOF M | Prepandemic:<br>Acinetobacter spp.: 109<br>E. coli: 343<br>Klebsiella spp.: 133<br>Proteus spp.: 93<br>Pseudomonas spp.: 128<br>CoNS: 128<br>S. aureus: 493<br>Streptococcus spp.: 281<br>Enterococcus spp.: 45<br>Other: 102<br><br>Pandemic:<br>Acinetobacter spp.: 283<br>E. coli: 168<br>Klebsiella spp.: 326<br>Proteus spp.: 138<br>Pseudomonas spp.: 133<br>CoNS: 256<br>S. aureus: 440<br>Streptococcus spp.: 192<br>Enterococcus spp.: 128<br>Other: 217 |
| 67 [86] | 2016 - 2020<br>Pre-pandemic | General | UTI: 1900<br>Wounds: 1566<br>BSI: 175<br>Other: 602 | Culture<br>Vitek 2 compact<br>Biochemical | Prepandemic:<br>Enterococcus spp.: 140<br>Staphylococcus spp.: 996<br>Klebsiella spp.: 357<br>Acinetobacter spp.: 24<br>Pseudomonas spp.: 320<br>E. coli: 1482<br>Proteus spp.: 216<br>Enterobacter spp.: 87<br>Salmonella spp.: 133<br>Other: 58<br><br>Pandemic:<br>Enterococcus spp.: 8<br>Staphylococcus spp.: 91<br>Klebsiella spp.: 41<br>Acinetobacter spp.: 4<br>Pseudomonas spp.: 46 |

| | | | | | | |
|---|---|---|---|---|---|---|
| 68 [87] | Jan 2017 – Dec Pre-pandemic | 2020 | ICU | BSI | Bact/ Alert 3D Vitek 2 compact | E. coli: 112 Proteus spp.: 22 Enterobacter spp.: 13 Salmonella spp.: 13 Other: 3 Prepandemic: S. aureus: 51 CoNS: 183 Group D strep.: 2 E. faecalis: 18 E. coli: 15 K. pneumoniae: 32 P. mirabillis: 5 P. aeruginosa: 15 A. baumanii: 31 NFB: 10 E. cloacae: 2 C. freundi: 3 P. stuartii: 1 Pandemic: S. aureus: 11 CoNS: 135 Group D strep.: 3 E. faecalis: 24 E. coli: 12 K. pneumoniae: 38 P. mirabillis: 3 P. aeruginosa: 15 A. baumanii: 14 NFB: 1 E. cloacae: 2 P. stuartii: 1 |
| 69 [88] | Jan - Jun pandemic | 2020 | General | Stool | Immunologic PCR | C. difficile: 50 |
| 70 [89] | Sep 2017 – Oct Pre-pandemic | 2021 | ICU Inf. Dis. | Rectal swabs UTI Sputum BSI Skin | Culture Vitek 2 MALDI TOF MS Biochemical | Klebsiella spp.: 53 E. coli: 11 |

| | | | | | | |
|---|---|---|---|---|---|---|
| 71 [90] | Oct 2019 – Oct Pre-pandemic | 2020 | General | UTI: 35<br>BSI: 17<br>Others: 20 | Bd Phoenix<br>Vitek 2 compact | Klebsiella spp.: 51<br>A. baumanii: 10<br>A. lwoffii: 1<br>P. aeruginosa: 10 |
| 72 [91] | Jan 2022 – Aug Post-pandemic | 2023 | Ophthalmology | Conjunctival secretion | Culture<br>Biochemical | S. aureus: 95<br>CoNS: 35<br>Group A strep.: 3<br>Enterococcus spp.: 4<br>Corynebacterium spp.: 2<br>Klebsiella spp.: 7<br>Proteus spp.: 6<br>P. aeruginosa: 3<br>E. coli: 2<br>Enterobacter spp.: 1<br>Haemophilus spp.: 2<br>S. marcescens: 1 |
| 73 [92] | Mar 2020 – Jan pandemic | 2021 | Inf. Dis. | Stool | ELFA | C. difficile |
| 74 [93] | Jan 2020 – Dec pandemic | 2021 | ICU | Puss<br>Catheters<br>Peritoneal fluid<br>TA<br>Sputum | PCR | E. coli: 14<br>K. pneumoniae: 32 |
| 75 [94] | Jan 2016 – Jun Pre-pandemic | 2022 | General | Wounds<br>Skin | NR | E. coli: 16<br>Klebsiella spp.: 12<br>Enterococcus spp.: 4<br>Pseudomonas spp.: 2<br>P mirabilis: 1<br>S. aures: 2<br>Streptococcus spp.: 1<br>Acinetobacter spp.: 1<br>Other: 1 |
| 76 [95] | Jan 2016 – Dec Pre-pandemic | 2021 | Cardiology | BSI | Culture<br>Vitek 2<br>PCR | S. aureus: 55<br>Enterococcus spp.: 33<br>CoNS: 18<br>Other strep.: 17<br>Viridans strep.: 10<br>Group D strep.: 9<br>P. aeruginosa: 9<br>H. influenzae: 6 |

| | | | | | | |
|---|---|---|---|---|---|---|
| | | | | | | E. coli: 4<br>K. pneumoniae: 4<br>Salmonella spp.: 3<br>Others: 3 |
| 77 [96] | Jan 2021 – Jun<br>pandemic | 2021 | Emergency | UTI: 27.64%<br>Wounds: 23.08%<br>BSI: 13.95%<br>Sputum: 9.65%<br>Abscess: 7.82%<br>Bile: 6.52%<br>BAL/ TA: 4.56%<br>Peritoneal fluid: 4.30%<br>Ascites: 0.78%<br>CSF: 0.52%<br>Catheters: 0.52%<br>Others: 0.65% | Vitek 2 compact<br>MALDI TOF MS | E. coli: 219<br>Klebsiella spp.: 124<br>S. aureus: 96<br>Enterococcus spp.: 85<br>CoNS: 79<br>Streptococcus spp.: 49<br>Pseudomonas spp.: 62<br>Proteus spp.: 37<br>Enterobacter spp.: 22<br>Acinetobacter spp.: 17<br>Citrobacter spp.: 17<br>Others: 51 |
| 78 [97] | Jan 2019 – Jun<br>Pre-pandemic | 2022 | Inf. Dis. | UTI | NR | K. pneumoniae: 76<br>P. stuartii: 4<br>E. cloacae: 3<br>E. coli: 1<br>Enterobacter spp.: 1<br>Citrobacter spp.: 1<br>S. marcescens: 1 |
| 79 [98] | 2019 -<br>Pre-pandemic | 2020 | GE | Biopsy<br>Stool | NR | H. pylori |
| 80 [99] | Mar - Dec<br>pandemic | 2020 | Int. Med.<br>Pneumology | BSI<br>UTI<br>Sputum<br>Stool | NR | Klebsiella spp.: 4<br>S. aureus: 1<br>E. coli: 2<br>C. difficile: 14 |
| 81 [100] | Jan 2019 – Feb<br>Mar 2020 – May<br>Pre-pandemic | 2020<br>2021 | GE<br>Inf. Dis.<br>Int. Med.<br>Surgery<br>ICU | Stool | Immunologic | C. difficile |
| 82 [101] | Jul 2020 – Jun<br>pandemic | 2022 | Pneumo-phtysiology | Sputum | Culture<br>PCR | TB |
| 83 [102] | Jan 2020 – Jan<br>pandemic | 2022 | General | Sputum: 166<br>BSI: 166<br>UTI: 84<br>Stool: 16 | Culture<br>PCR | S. aureus: 37<br>S. pneumoniae: 21<br>S. pyogenes: 18<br>E. faecalis: 12 |

| | | | | | |
|---|---|---|---|---|---|
| | | | | | E. coli: 33<br>Klebsiella spp.: 44<br>P. aeruginosa: 37<br>C. difficile: 14<br>Others: 13 |
| 84 [103] | Jul 2021<br>pandemic | Inf. Dis. | Sputum | NR | TB, S. liquefaciens, K. pneumoniae, C. difficile |
| 85 [104] | 2020 - 2021<br>pandemic | Neurology, D-V | BSI, CSF | Immunologic<br>Serologic | T. pallidum |
| 86 [105] | Jan 2022 –<br>Sept 2023<br>Post-pandemic | ICU<br>Pneumology<br>Pediatrics | Respiratory | NR | S. pneumoniae: 1<br>H. influenzae: 1 |
| 87 [106] | Sep - Dec 2018<br>Sep - Dec 2020<br>Sep - Dec 2022<br>Pre-pandemic | Urology | UTI | Culture<br>Biochemical | Prepandemic:<br>E. coli: 253<br>Klebsiella spp.: 78<br>Proteus spp.: 34<br>Pseudomonas spp.: 14<br>Enterococcus spp.: 84<br>Staphylococcus spp.: 15<br><br>Pandemic:<br>E. coli: 365<br>Klebsiella spp.: 208<br>Proteus spp.: 47<br>Pseudomonas spp.: 28<br>Enterococcus spp.: 80<br>Staphylococcus spp.: 23 |

Table S4. Antibiotic use as treatment and methodology details for antibiotic susceptibility testing

| No. | Period | Antibiotics used as therapy | AST method | AST protocol |
|---|---|---|---|---|
| 1 [20] | Jan 2021<br>pandemic | Ceftriaxone | Empiric | NR |
| 2 [21] | Jan 2018 - Dec 2022<br>Pre-pandemic | NR | Vitek 2 compact, Micronaut-AM | EUCAST |
| 3 [22] | April 2022<br>pandemic | cefoperazone/sulbactam + gentamicin<br>teicoplanin + azithromycin | Vitek 2 compact | CLSI |
| 4 [23] | Jan - Dec 2022<br>pandemic | NA | NR | NR |

| | | | | |
|---|---|---|---|---|
| 5 [24] | Jan 2014 - Jan 2024<br>Pre-pandemic | Azythomycin +/- Rifampicin, Doxycycline, Gentamicin | Empiric | NA |
| 6 [25] | Jul - Dec 2021<br>pandemic | NR | Vitek 2 compact | NR |
| 7 [26] | Nov 2020 - Apr 2021<br>pandemic | NA | Vitek 2 compact | EUCAST |
| 8 [27] | 2002 - 2022<br>Pre-pandemic | surgery + penicillin/ amoxicillin 4-6 weeks | Empiric | NA |
| 9 [28] | Sep - Nov 2021<br>pandemic | Macrolides: 5<br>3rd cephalosporins: 171<br>Carbapenems: 30<br>Combination penicilins: 14<br>Fluoroquinolones: 2<br>Linezolid: 6<br>Glycopeptides: 7<br>Aminoglycosides: 1,<br>Colistin: 3<br>Doxycycline: 5<br>Cotrimoxazole: 2 | DD, Microscan | EUCAST |
| 10 [29] | Jan 2017 - Dec 2022<br>Pre-pandemic | NR | NR | NR |
| 11 [30] | Jan 2018 - Dec 2021<br>Pre-pandemic | Cephalosporins: 33.5%<br>Penicillin: 31.2%<br>Glycopeptides: 28.7%<br>Amynoglycosides: 2.4%<br>Macrolides: 2.3%<br>Quinolones: 1.1%<br>Tetracycline: 0.4%<br>Carbapenems: 0.2%<br>Nitroimidazoles: 0.2% | Empiric | NR |
| 12 [31] | Jan - May 2021<br>pandemic | Penicilins: 85<br>Cephalosporins: 163<br>Carbapenems: 79<br>Quinolones: 51<br>Linezolid: 27<br>Cyclines: 23<br>Glycopeptides: 19<br>Aminoglycosides: 10<br>Macrolides: 3<br>Colistin: 11 | Empiric | NR |

| | | | | |
|---|---|---|---|---|
| | | Cotrimoxazole: 23<br>Metronidazole: 11<br>Other: 8 | | |
| 13 [32] | May 2021 - Aug 2023<br>Post-pandemic | NA | E-test | EUCAST |
| 14 [33] | 2016 - 2020<br>Pre-pandemic | NA | DD | CLSI |
| 15 [34] | Oct - Dec 2020<br>Oct - Dec 2021<br>pandemic | Ward:<br>Ceftraixone: 100<br>Imipenem: 15<br>Meropenem: 6<br>Linezolid: 5<br>ICU:<br>Imipenem/ colstatin: 25<br>Ceftriaxone: 13<br>Meropenem: 1<br>Pieperacillin/ tazo: 1<br>Linezolid: 1 | DD | EUCAST |
| 16 [35] | Nov 2021 - Jan 2022<br>Pre-pandemic | Azythromycin: 36<br>Ceftriaxone: 63<br>Doxycycline: 9<br>Linezolid: 7<br>Carbapemens: 6<br>Cotrimoxazole: 4<br>Macrolides: 3<br>Tigecycline: 2<br>Glycopeptides: 2<br>Rifaximin: 3<br>Penicilin combinations: 12 | NR | NR |
| 17 [36] | Jan 2017 - Dec 2022<br>pandemic | NA | Sensititre, Micronaut | EUCAST |
| 18 [37] | May - Dec 2021<br>pandemic | NA | Vitek 2 compact, DD | EUCAST |
| 19 [38] | 2011 - 2020<br>Pre-pandemic | NA | NR | NR |
| 20 [39] | Jan 2016 - Oct 2022<br>Pre-pandemic | NA | Vitek 2 compact | EUCAST |
| 21 [40] | Oct - Nov 2021<br>pandemic | NR | NR | NR |

| | | | | |
|---|---|---|---|---|
| 22 [41] | Jan 2022 - Dec 2023<br>Post-pandemic | NA | Vitek 2 compact | CLSI |
| 23 [42] | Oct 2020 - May 2021<br>pandemic | NR | Vitek 2 compact | CLSI |
| 24 [43] | May 2021<br>pandemic | meropenem, linezolid, teicoplanin | Empiric | NA |
| 25 [44] | Jan 2022 - Dec 2022<br>pandemic | NA | MicroScan | CLSI |
| 26 [45] | Jan 2016 - Dec 2022<br>Pre-pandemic | NR | NR | NR |
| 27 [46] | Sep 2022 - Feb 2023<br>Post-pandemic | NR | NR | NR |
| 28 [47] | NR<br>pandemic | Meropenem: 6<br>Levofloxacin: 1<br>Linezolid: 1<br>Ceftriaxone: 2<br>Doxycycline: 2<br>Colistin: 1 | Vitek 2 compact, DD | EUCAST |
| 29 [48] | Jan 2022 - Oct 2023<br>Post-pandemic | NA | Vitek 2 compact, DD, E-test, microdillution, immunochromatographic tests, PCR | EUCAST |
| 30 [49] | Mar - Dec 2020<br>pandemic | NR | NR | NR |
| 31 [50] | Jan 2010 - Dec 2022<br>Pre-pandemic | isoniazid, rifampin, pyrazinamide, and ethambutol | NR | NR |
| 32 [51] | Jan 2019 - Dec 2020<br>Pre-pandemic | NA | NR | NR |
| 33 [52] | Sep 2020 - Apr 2021<br>pandemic | Vancomycin: 31<br>Metronidazole: 9<br>Rifaximin: 17 | NR | NR |
| 34 [53] | Sep 2019 - Sep 2022<br>Pre-pandemic | Vancomycin: 140<br>Metronidazole: 55 | NR | NR |
| 35 [54] | Mar - Jun 2020<br>pandemic | NR | NR | NR |
| 36 [55] | Aug 2020 - Aug 2023<br>Post-pandemic | NA | Vitek 2 | CLSI |
| 37 [56] | 2019 - 2023<br>Pre-/ Post-pandemic | NA | Vitek 2 | CLSI |

| | | | | |
|---|---|---|---|---|
| 38 [57] | 2017 - 2022<br>Pre-pandemic | Amoxicillin: 15<br>Metronidazole: 15<br>Piperacillin/ tazo: 19 | NA | NA |
| 39 [58] | 2021 - 2022<br>pandemic | NA | NR | NR |
| 40 [59] | Oct 2020 - Jun 2022<br>pandemic | NA | DD, microdilution | NR |
| 41 [60] | Apr 2020 - March 2022<br>pandemic | cephalospirns: 21<br>carbapenems: 11<br>fluoroquinolones: 5<br>betalactamines: 6<br>aminoglicosides: 5 | NR | NR |
| 42 [61] | Apr 2020 - Jun 2021<br>pandemic | NA | Vitek 2 compact | NR |
| 43 [62] | Jan - Jul 2021<br>pandemic | NA | Chromogenic culture, PCR | NA |
| 44 [63] | Mar - Dec 2020<br>pandemic | vancomycin: 447<br>fidaxomycin: 2 | NR | NR |
| 45 [64] | Sep 2018 - Feb 2019<br>Sep 2020<br>Feb 2021<br>Pre-pandemic | NA | DD | CLSI, EUCAST |
| 46 [65] | Jan 2015 - Dec 2021<br>Pre-pandemic | NR | NR | NR |
| 47 [66] | Jan 2017 - Jul 2022<br>Pre-pandemic | NA | DD, Vitek 2 compact, PCR | EUCAST |
| 48 [67] | Oct 2018 - May 2022<br>Pre-pandemic | NA | DD, mcirodillution, MicroScan | EUCAST |
| 49 [68] | 2017 - 2021<br>Pre-pandemic | NR | NR | NR |
| 50 [69] | Jan 2019 - Apr 2020<br>Pre-pandemic | 2HRZE + 4HR | NR | NR |
| 51 [70] | Jan 2019 - Dec 2021<br>Pre-pandemic | NA | Vitek 2 compact, Micronaut-AM | EUCAST |
| 52 [71] | Sep 2019 - May 2022<br>Pre-pandemic | NA | DD | CLSI |
| 53 [72] | Jan 2020 - Mar 2022<br>Pre-pandemic | vancomycin: 86<br>metronidazole: 30 | NA | NA |

| | | | | |
|---|---|---|---|---|
| 54 [73] | Mar 2017 - Feb 2018<br>Mar 2020 - Feb 2021<br>Pre-pandemic | metronidazole: 16<br>vancomycin: 144<br>tigecycline: 27 | NA | NA |
| 55 [74] | Feb 2019 - Ju 2020<br>Pre-pandemic | amoxicillin: 111<br>amoxi/ clav: 5<br>clarythromycin: 49<br>metronidazole: 67 | NR | empiric |
| 56 [75] | 2017 - 2022<br>Pre-pandemic | NR | NR | NR |
| 57 [76] | Jun 2018 - Jun 2020<br>Pre-pandemic | NA | Vitek 2 | CLSI |
| 58 [77] | Mar - Nov 2018<br>Mar - Nov 2019<br>Mar - Nov 2020<br>Pre-pandemic | NR | NR | NR |
| 59 [78] | Mar 2020 - Nov 2022<br>pandemic | NA | Vitek 2 compact, Micronaut, DD, E-test | EUCAST |
| 60 [79] | 2005 - 2022<br>Pre-pandemic | 9 HIN<br>MTX | NR | NR |
| 61 [80] | 2021<br>pandemic | ceftriaxone, linezolid<br>ampicillin, vancomycin | DD, E-test | EUCAST |
| 62 [81] | Mar 2020 - Aug 2022<br>pandemic | NA | MicroScan Walkaway DxM1040 | CLSI |
| 63 [82] | 1990 - 2021<br>Pre-pandemic | cephalosporins,<br>aminoglycosies,carbapenems,s,s,<br>glycoeptides | NR | NR |
| 64 [83] | Apr 2020 - Dec 2020<br>pandemic | azythromicycin: 26<br>vancomycin: 78<br>metronidazole: 50<br>Tigecycline: 35<br>Rifaximin: 17 | Vitek 2 compact | EUCAST |
| 65 [84] | Jul 2021<br>pandemic | ampicillin, cotrimoxazole | NR | NR |
| 66 [85] | Apr 2019 - Mar 2020<br>Jul 2021 - Jun 2022<br>Pre-pandemic | NA | Vitek 2 compact | CLSI |
| 67 [86] | 2016 - 2020<br>Pre-pandemic | NA | DD, Vitek 2 compact | CLSI |
| 68 [87] | Jan 2017 - Dec 2020<br>Pre-pandemic | NA | DD, Vitek 2 compact | CLSI |

| | | | | |
|---|---|---|---|---|
| 69 [88] | Jan - Jun 2020<br>pandemic | NR | NA | NA |
| 70 [89] | Sep 2017 - Oct 2021<br>Pre-pandemic | Ceftazidim/ avibactam: 6<br>Ceftolozane/ tazo: 1<br>Imipenem/ cilastatin/ relebactam: 1<br>Colistin: 7<br>Carbapenems: 25<br>Cephalosporins: 14<br>Piper/ tazo: 2<br>Quinolones: 19<br>Vancomycin: 7<br>Linezolid: 10<br>Amikacin: 10<br>Doxycycline: 6 | NR | EUCAST |
| 71 [90] | Oct 2019 - Oct 2020<br>Pre-pandemic | NA | Bd Phoenix, Vitek 2 compact, DD | CLSI |
| 72 [91] | Jan 2022 - Aug 2023<br>Post-pandemic | NA | microdilution | CLSI |
| 73 [92] | Mar 2020 - Jan 2021<br>pandemic | NR | NA | NA |
| 74 [93] | Jan 2020 - Dec 2021<br>pandemic | NA | DD, Vitek 2 | CLSI |
| 75 [94] | Jan 2016 - Jun 2022<br>Pre-pandemic | Metronidazole: 36<br>Carbapenems: 20<br>Vancomycin: 15<br>Quinolones: 8<br>Cephalosporins: 7<br>Piper/ tazo: 5<br>Gentamicin: 2 | DD, empiric | NR |
| 76 [95] | Jan 2016 -Dec 2021<br>Pre-pandemic | NA | DD | NR |
| 77 [96] | Jan 2021 - Jun 2021<br>pandemic | NA | Vitek 2 compact | CLSI |
| 78 [97] | Jan 2019 - Jun 2022<br>Pre-pandemic | NA | DD, Microscan, biochemical, immunologic | EUCAST |
| 79 [98] | 2019 - 2020<br>Pre-pandemic | amoxicillin: 54<br>metronidazole: 26<br>clarythromycin: 36 | NA | NA |
| 80 [99] | Mar - Dec 2020<br>pandemic | NR | NR | NR |

| | | | | |
|---|---|---|---|---|
| 81 [100] | Jan 2019 - Feb 2020<br>Mar 2020 - May 2021<br>Pre-pandemic | NR | NR | NR |
| 82 [101] | Jul 2020 - Jun 2022<br>pandemic | HINZ | NR | NR |
| 83 [102] | Jan 2020 - Jan 2022<br>pandemic | cephalosporins: 41.4%<br>macrolides: 23.2%<br>penicillin: 19.7%<br>aminoglycosides: 7.4%<br>Tetracycline: 3.9%<br>Quinolones: 3.4%<br>Carbapenems: 0.2%<br>glycopeptides: 0.2%<br>nitroimidazole: 0.6% | NR | NR |
| 84 [103] | Jul 2021<br>pandemic | Meropenem, Vancomycin, Moxifloxacin, Colistin, Cotrimoxazole, HINZE | NR | NR |
| 85 [104] | 2020 - 2021<br>pandemic | Penicillin | NR | NR |
| 86 [105] | Jan 2022 - Sept 2023<br>Post-pandemic | NR | NR | NR |
| 87 [106] | Sep - Dec 2018<br>Sep - Dec 2020<br>Sep - Dec 2022<br>Pre-pandemic | NA | DD | CLSI |

Table S5. Antibiotic susceptibility testing results and phenotypes

| No. | Period | Antibiotics resistance | Resistance phenotype |
|---|---|---|---|
| 1 [20] | Jan 2021<br>pandemic | NA | NA |
| 2 [21] | Jan 2018 - Dec 2022<br>Pre-pandemic | E. coli: Ampicillin, Cotrimoxazole (high)<br>Klebsiella spp.: Amoxicillin/Clavulanate, Ceftazidime, Cefepime, Gentamicin, Cotrimoxazole (high); Colistin - 15%<br>Enterococcus spp.: Ampicillin, Ciprofloxacin, Gentamicin, Streptomycin, Teicoplanin (high)<br>P. aeruginosa: Amikacin, Ceftazidime, Cefepime, Imipenem, Meropenem (high); Colistin - 4%<br>A. baumannii: Gentamicin, Ceftazidime, Cefepime, Imipenem, Meropenem, Amikacin (high); Colistin - 20% | E. coli: ESBL: 13.90%, CRE: 0.20%, MDR: 33.30%<br>Klebsiella spp.: ESBL: 32.90%, CRE: 10.20%, MDR: 48%<br>E. faecalis: VRE: 2%<br>E. faecium: VRE: 37%<br>P. aeruginosa: MDR: 37%<br>A. baumanii: MDR: 87% |

| | | | |
|---|---|---|---|
| 3 [22] | April 2022<br>pandemic | Rhizobium radiobacter:<br>Tigecycline – 100%, Colistin – 100% | NR |
| 4 [23] | Jan - Dec 2022<br>pandemic | NA | NA |
| 5 [24] | Jan 2014 - Jan 2024<br>Pre-pandemic | NR | NR |
| 6 [25] | Jul - Dec 2021<br>pandemic | NR | Unspecified MDR: 85.84%<br>MRSA: 42.31% |
| 7 [26] | Nov 2020 - Apr 2021<br>pandemic | Enterococcus spp.:<br>Ampicillin: 53.06%<br>Gentamicin: 63.27%<br>Streptomycin: 63.27%<br>Ciprofloxacin: 68.37%<br>Erythromycin: 100.00%<br>Linezolid: 12.24%<br>Teicoplanin: 27.55%<br>Vancomycin: 36.73%<br>Tetracycline: 89.80%<br>Tigecycline: 2.04% | vanA: 27<br>vanB: 7<br>vanC: 2 |
| 8 [27] | 2002 - 2022<br>Pre-pandemic | NA | NA |
| 9 [28] | Sep - Nov 2021<br>pandemic | NR | Unspecified GNB MDR: 33.33%, ESBL: 11.11%, CRE: 5.56%<br>MRSA: 100% |
| 10 [29] | Jan 2017 - Dec 2022<br>Pre-pandemic | NR | NR |
| 11 [30] | Jan 2018 - Dec 2021<br>Pre-pandemic | NR | Unspecified MDR: 37.02% |
| 12 [31] | Jan - May 2021<br>pandemic | NR | NR |
| 13 [32] | May 2021 - Aug 2023<br>Post-pandemic | H. pylori:<br>Claithromycin: 50.72%<br>Quinolones: 21.74%<br>Metronidazole: 31.88%<br>Amoxicillin: 1.45%<br>Rifampicin: 4.35%<br>Tetracycline: 1.45% | H. pylori MDR: 24.64%, CH+FQ+MET: 21.74, CH+MET+RIF:1.45%, AMX+CH+RIF: 1.45% |
| 14 [33] | 2016 - 2020<br>Pre-pandemic | GNB: Aminopenicillins, 2nd Cephalosporins, Nalidixic Acid, Cotrimoxazole (high) | NR |

| | | | |
|---|---|---|---|
| | | Other GN: Cephalosporins, Gentamicin, Ticarcillin/Clavulanate (high), Imipenem, Piperacillin/Tazobactam, Tobramycin, Penicillin (up)<br>GP: Clindamycin, Erythromycin, Gentamicin, Tetracycline (high), Penicillin (up) | |
| 15 [34] | Oct - Dec 2020<br>Oct - Dec 2021<br>pandemic | NR | MDR:<br>P. aeruginosa: 20.00%<br>A. baumanii: 33.33%<br>K. pneumoniae: 100%<br>S. aureus: 100%<br>Proteus spp.: 100%<br><br>XDR:<br>P. aeruginosa: 80.00%<br>A. baumanii: 66.66% |
| 16 [35] | Nov 2021 - Jan 2022<br>Pre-pandemic | NR | NR |
| 17 [36] | Jan 2017 - Dec 2022<br>pandemic | Oxacillin: 39.11%<br>Penicillin: 87.24%<br>Clindamycin: 36.06%<br>Erythromycin: 49.97%<br>Gentamicin: 5.95%<br>Tetracycline: 45.01%<br>Ciprofloxacin: 9.98%<br>Moxifloxacin: 7.90%<br>Rifampicin: 5.32%<br>Cotrimoxazole: 0.96%<br>Linezolid: 0.12%<br>Teicoplanin: 0.18%<br>Vancomycin: 0.00% | MRSA: 39.11% |
| 18 [37] | May - Dec 2021<br>pandemic | Penicillin: 3.29%<br>Erythromycin: 25.00%<br>Clindamycin: 23.68%<br>Linezolid: 1.97% | MDR Group B Streptococcus: 0% |
| 19 [38] | 2011 - 2020<br>Pre-pandemic | NR | MRSA: 5.20%<br>MDR/ CRE P. aeruginosa: 5.50%<br>MDR A. baumanii: 5.20% |
| 20 [39] | Jan 2016 - Oct 2022<br>Pre-pandemic | CoNS: Penicillin - 23.73%, Oxacillin - 23.73%, Tetracycline - 25.42%, Gentamicin - 20.34%, Quinolones - 6.78%<br>S. aureus: Penicillin - 19.63%, Cefoxitin - 13.08%, Clindamycin - 17.76%, Erythromycin - 16.82%, Oxacillin - 13.08%, Tetracycline - 19.63% | Unspecified MDR: 70.59%, ESBL: 14.71%<br>MDR:<br>P. aeruginosa: 100%<br>E. coli: 83.33%<br>A. baumanii: 66.66% |

| | | | |
|---|---|---|---|
| | | E. faecalis: Gentamicin - 18.18%, Streptomycin - 13.64%, Tetracycline - 27.27%, Clindamycin - 27.27%, Ciprofloxacin - 13.64%<br>E. coli: Gentamicin - 20.83%, Cefotaxime - 16.67%, Ceftazidime - 16.67%, Cefepime - 16.67%, Nitrofurantoin - 25.00%, Cotrimoxazole - 4.17%<br>Enterobacter spp.: Cephalosporins - 19.05%, Gentamicin - 28.57%, Ciprofloxacin - 14.29%, Amoxicillin/Clavulanate - 38.10%<br>P. mirabilis: Penicillin combinations - 40.00%, Imipenem - 60.00%<br>P. aeruginosa: Amikacin - 21.05%, Combination - 21.05%, Ceftazidime - 21.05%, Imipenem - 15.79%, Ciprofloxacin - 21.05%<br>A. baumannii: Cefazolin - 33.33%, Cefepime - 22.22%, Ciprofloxacin - 22.22%, Piperacillin - 22.22%<br>R. picketti: Ticarcillin - 16.67%, Amikacin - 25.00%, Tobramycin - 25.00%, Colistin - 33.33% | Enterobacter spp.: 50.00%<br>R. picketti: 75.00%<br>MRSA: 66.67%, MRCoNS: 89.47%, |
| 21 [40] | Oct - Nov 2021 pandemic | NR | NR |
| 22 [41] | Jan 2022 - Dec 2023 Post-pandemic | CoNS: Clindamycin - 33.90%, Erythromycin - 49.12%, Gentamicin - 41.07%, Penicillin - 77.97%, Tetracycline - 63.79%, Linezolid - 3.64%<br>S. aureus: Ciprofloxacin - 33.90%, Clindamycin - 40.00%, Erythromycin - 70.49%, Gentamicin - 43.55%, Penicillin - 80.65%, Tetracycline - 66.10%, Tigecycline - 43.48%, Linezolid - 3.51%<br>S. pneumoniae: Amoxicillin - 68.75%, Erythromycin - 43.75%, Ofloxacin - 37.50%, Penicillin - 81.25%, Cotrimoxazole - 75.00%, Tetracycline - 43.75%<br>S. viridans: Clindamycin - 33.33%, Erythromycin - 33.33%, Oxacillin - 75.00%, Vancomycin - 50.00%<br>E. coli: Amoxicillin/Clavulanic Acid - 33.33%, Ampicillin - 75.00%, Aztreonam - 40.00%, Moxifloxacin - 50.00%, Ofloxacin - 100.00%, Piperacillin/Tazobactam - 33.33%, Cotrimoxazole - 75.00%, Tetracycline - 100.00%<br>K. pneumoniae: Amoxicillin/Clavulanic Acid - 76.92%, Ampicillin - 100.00%, Aztreonam - 33.33%, Cefuroxime - 53.85%, Cefpirome - 50.00%, Ceftazidime - 35.71%, Gentamicin - 54.55%, Tobramycin - 54.85%, Moxifloxacin - 37.50%, Meropenem - 50.00%, Cotrimoxazole - 33.33%, Tetracycline - 100.00%, Tigecycline - 41.67%, Colistin - 7.69%<br>Citrobacter spp.: Amoxicillin/Clavulanic Acid - 100.00%, Ampicillin - 100.00%, Aztreonam - 33.33%, Cefpirome - 50.00%, Cefotaxime - 50.00%, Tobramycin - 50.00%, Ciprofloxacin - 50.00%, Ofloxacin - 50.00%, Imipenem - 33.33%, Tetracycline - 100.00%, Chloramphenicol - 50.00%, Tigecycline - 66.67%, Colistin - 33.33%<br>Proteus spp.: Ampicillin - 50.00%, Tobramycin - 33.33%, Imipenem - 50.00%, Tetracycline - 50.00%, Chloramphenicol - 33.33%, Colistin - 66.67% | NR |

| | | | |
|---|---|---|---|
| | | P. aeruginosa: Amoxicillin/Clavulanic Acid - 66.67%, Cefuroxime - 55.56%, Cefpirome - 40.00%, Gentamicin - 58.33%, Ertapenem - 41.67%, Cotrimoxazole - 81.82% | |
| 23 [42] | Oct 2020 - May 2021<br>pandemic | NR | Unspecified MDR: 50.42%, ESBL: 40.34%, CRE: 36.13%, XDR: 31.09%<br>VRE: 13.33%, MRSA: 66.67%, MRCoNS: 80.65% |
| 24 [43] | May 2021<br>pandemic | NA | NA |
| 25 [44] | Jan 2022 - Dec 2022<br>pandemic | E. coli: Ampicillin - 67.73%, Cefotaxime - 44.59%, Cotrimoxazole - 40.81%<br>S. aureus: Tetracycline - 44.44%, Erythromycin - 35.35%<br>MRSA: Tetracycline - 83.67%, Erythromycin - 83.33%, Clindamycin - 73.01%<br>Klebsiella spp.: Cefotaxime - 73.85%, Cefuroxime - 68.39%, Cefalexin/Cefazolin - 67.81%, Ceftazidime - 66.09%, Cefepime - 60.34%, Cotrimoxazole - 54.59%, Aminopenicillins - 45.40%, Gentamicin - 43.10%, Ciprofloxacin - 37.35%, Fosfomycin - 33.33%<br>Pseudomonas spp.: Gentamicin - 33.33%<br>E. faecalis: Rifampicin - 42.47%<br>E. faecium: Ampicillin - 89.47%, Gentamicin - 73.68%, Ciprofloxacin - 92.10%, Levofloxacin - 86.84%, Rifampicin - 89.47%<br>Other: NR | E. coli: ESBL: 49.56%<br>Klebsiella spp.: MDR: 43.68%, XDR: 19.74%<br>MRSA: 55.75%<br>other: NR |
| 26 [45] | Jan 2016 - Dec 2022<br>Pre-pandemic | P. aeruginosa: Ceftriaxone (high), Ceftazidime (high), Ciprofloxacin (high), Levofloxacin (high), Meropenem (high), Piperacillin (high), Ticarcillin (high), Tobramycin (high)<br>S. aureus: Cefuroxime (high), Clindamycin (high), Oxacillin (high)<br>A. baumannii: Ceftazidime (high), Cefepime (high), Ciprofloxacin (high)<br>Klebsiella spp.: Cotrimoxazole (high)<br>E. coli: Levofloxacin (high), Cotrimoxazole (high)<br>Group B Streptococcus: Clindamycin, Erythromycin, Cotrimoxazole<br>Salmonella spp.: Clindamycin, Erythromycin<br>Morganella spp.: Cotrimoxazole | NR |
| 27 [46] | Sep 2022 - Feb 2023<br>Post-pandemic | NR | NR |
| 28 [47] | NR<br>pandemic | K. pneumoniae:<br>Ampicillin: 100.00%<br>Amoxi/Clav: 100.00%<br>Piper/Tazo: 100.00%<br>Cefotaxime: 100.00%<br>Ceftazidime: 100.00%<br>Cefepime: 100.00% | CRE: 100%<br>KPC: 88.89%, OXA-48: 55.56% |

| | | | |
|---|---|---|---|
| 29 [48] | Jan 2022 - Oct 2023<br>Post-pandemic | Carbapenems: 100.00%<br>Amikacin: 55.56%<br>Gentamicin: 77.78%<br>Ciprofloxacin: 100.00%<br>Fosfomycin: 88.89%<br>Cotrimoxazole: 66.67%<br>Colistin: 62%<br>Fosfomycin: 67%<br>Amikacin: 78%<br>Amoxi/ Clav:100%<br>Ampicillin: 100%<br>Aztreonam: 99%<br>Cefepime: 100%<br>Ceftazidime/ Avibactam: 64%<br>Ceftriaxone: 100%<br>Cefuroxime: 100%<br>Ciprofloxacin: 100%<br>Gentamicin: 85%<br>Imipenem: 96%<br>Meropenem: 99%<br>Piper/ Tazo: 100%<br>Cotrimoxazole: 89%<br>Norfloxacin: 100%<br>Tobramycin: 100% | Unspecified CRE: 100%<br>KPC: 32, OXA-48: 19.75%, NDM: 9.88%,<br>NDM+OXA-48-like: 49.38% |
| 30 [49] | Mar - Dec 2020<br>pandemic | NR | NR |
| 31 [50] | Jan 2010 - Dec 2022<br>Pre-pandemic | NR | MDR: 0 |
| 32 [51] | Jan 2019 - Dec 2020<br>Pre-pandemic | Fluoroquinolones: 37.6%<br>Aminoglycosides: 34%<br>Carbapenems: 6.1% | Unspecified MDR: 29.96%<br>MDR Pseudomonas spp.: 67.65%<br>MDR Klebsiella spp.: 47.62%<br>MDR Enterococcus spp.: 50.00%<br>MDR E. coli: 22.60%<br>MDR Proteus: 29.41%<br>MDR Staphylococcus: 12.50% |
| 33 [52] | Sep 2020 - Apr 2021<br>pandemic | NR | NR |
| 34 [53] | Sep 2019 - Sep 2022<br>Pre-pandemic | NR | NR |

| | | | |
|---|---|---|---|
| 35 [54] | Mar - Jun 2020<br>pandemic | NR | NR |
| 36 [55] | Aug 2020 - Aug 2023<br>Post-pandemic | Ampi/Sulbactam - 33.33%<br>Piperacillin/Tazo - 20.06%<br>Fluoroquinolones - 15.75%<br>Carbapenems - 21.24%<br>Cephalosporins - 21.63%<br>Aminoglycosides - 3.28%<br>Ticar/Clav - 32.18%<br>Piperacillin - 55.78% | Unspecified MDR: 27.48%, ESBL: 17.00%, CRE: 6.23%<br>MRSA: 20.00%, VRE: 11.30% |
| 37 [56] | 2019 - 2023<br>Pre-/ Post-pandemic | Amoxicillin - 12.24%<br>Nitrofurantoin - 14.88%<br>Ampicillin/Sul - 12.14%<br>Macrolides - 13.51%<br>Fosfomycin - 18.62%<br>Piper/Tazo - 10.79%<br>Carbapenems - 11.45%<br>Glycopeptides - 8.96%<br>Cephalosporins - 12.85%<br>Ticar/Clav - 20.86%<br>Piperacillin - 10.19% | Unspecified MDR: 16.79%, ESBL: 12.04%, CRE: 2.19%<br>MRSA: 9.09%, VRE: 26.09% |
| 38 [57] | 2017 - 2022<br>Pre-pandemic | NA | NA |
| 39 [58] | 2021 - 2022<br>pandemic | E. faecium: Amoxi/Clav - 42.86%, Cefuroxime - 71.43%, Ceftriaxone - 57.14%, Ciprofloxacin - 57.14%, Erythromycin - 42.86%<br>S. aureus: Penicillin - 47.41%<br>K. pneumoniae: Ticar/Clav - 48.48%, Piper/Tazo - 51.52%<br>A. baumannii: Amoxi/Clav - 50.00%, Oxacillin - 37.50%, Penicillin - 37.50%, Cefuroxime - 37.50%, Ciprofloxacin - 37.50%, Gentamicin - 37.50%<br>P. aeruginosa: Cefuroxime - 37.25%<br>Enterobacter spp.: Amoxi/Clav - 52.38%, Ciprofloxacin - 33.33%, Erythromycin - 33.33% | Unspecified MDR: 66.04%<br>MRSA: 20.00%, VRE: 14.29%, |
| 40 [59] | Oct 2020 - Jun 2022<br>pandemic | S. aureus: Penicillin - 19.37%, Oxacillin - 19.37%, Erythromycin - 14.66%, Clindamycin - 14.66%<br>E. coli: Ampicillin - 7.14%, Ceftazidime - 7.14%, Ceftriaxone - 7.14%<br>Klebsiella spp.: Ampicillin - 5.95%, Amoxi/Clav - 3.57%, Ceftriaxone - 3.57%<br>Enterococcus spp.: Ciprofloxacin - 3.70%, Chloramphenicol - 2.47%, Erythromycin - 3.70%, Levofloxacin - 3.70% | ESBL: Klebsiella spp.: 5.95%, E. coli: 3.70%, Serratia spp.: 33.33%<br>MRSA: 19.37%<br>HLAR Enterococcus: 3.70% |
| 41 [60] | Apr 2020 - March 2022<br>pandemic | NR | NR |

| | | | |
|---|---|---|---|
| 42 [61] | Apr 2020 - Jun 2021 pandemic | NR | Unspecified MDR: 64.63%<br>MRSA: 63.64%<br>Others: CD |
| 43 [62] | Jan - Jul 2021 pandemic | NA | vanA: 83.54%<br>vanB: 10.13%<br>other: 94.94% |
| 44 [63] | Mar - Dec 2020 pandemic | NR | NR |
| 45 [64] | Sep 2018 - Feb 2019<br>Sep 2020<br>Feb 2021<br>Pre-pandemic | Prepandemic: Staphylococcus spp.: Penicillin - 45.45%<br><br>Pandemic: Klebsiella spp.: Amoxi/Clav - 38.79%, Levofloxacin - 35.51%<br>Pseudomonas spp.: Amikacin - 51.61%, Aztreonam - 41.93%, Ceftazidime - 67.74%, Fosfomycin - 67.74%, Imipenem - 77.41%, Levofloxacin - 64.51%, Nitrofurantoin - 51.61%<br>Proteus spp.: Imipenem - 33.33%<br>Enterococcus spp.: Levofloxacin - 35.10%<br>Staphylococcus spp.: Penicillin - 47.36% | Prepandemic VRE: 1.57%<br>Pandemic VRE: 2.13%<br>Other: CD |
| 46 [65] | Jan 2015 - Dec 2021 Pre-pandemic | NR | NR |
| 47 [66] | Jan 2017 - Jul 2022 Pre-pandemic | Prepandemic: Levofloxacin - 75.30%, Imipenem - 53.63%, Meropenem - 55.40%, Piper/Tazo - 28.27%, Ceftazidime - 51.90%, Amikacin - 43.13%<br><br>Pandemic: Levofloxacin - 57.76%, Imipenem - 50.73%, Meropenem - 40.03%, Piper/Tazo - 52.03%, Ceftazidime - 55.43%, Amikacin - 44.73% | PDR Pseudomonas spp. (w/out colistin): 2.11% |
| 48 [67] | Oct 2018 - May 2022 Pre-pandemic | Colistin:<br>P.aeruginosa – 6.71%<br>A. Baumanii – 27.14%<br>Klebsiella spp. – 80.00%<br>Other: NR | MDR:<br>P. aeruginosa: 93.29%<br>A. baumanii: 91.43%<br>Klebsiella spp.: 31.25%<br>E. coli: 5.71%<br>Other GNB: 30%<br>MRSA: 30.68% |
| 49 [68] | 2017 - 2021 Pre-pandemic | NR | MRSA: 100%<br>Others: CD |
| 50 [69] | Jan 2019 - Apr 2020 Pre-pandemic | NR | NR |
| 51 [70] | Jan 2019 - Dec 2021 Pre-pandemic | Total: Aminoglycosides - 39.16%, Cephalosporins - 54.81%, Carbapenems - 34.63%, Quinolones - 54.22%, Cotrimoxazole - 41.23%, Colistin - 26.95%, Tigecycline - 2.05%, Fosfomycin - 35.48% | Total period MDR K. pneumoniae: 51.84%, CRE: 32.86%, MBL: 26.76%<br>Pandemic period MDR K. pneumoniae: 54.77%, CRE: 46.77%, MBL: 40.30% |

| | | | |
|---|---|---|---|
| | | Pandemic: Aminoglycosides - 50.69%, Cephalosporins - 60.48%, Carbapenems - 50.00%, Quinolones - 60.00%, Cotrimoxazole - 35.64%, Colistin - 43.78%, Tigecycline - 3.78%, Fosfomycin - 41.35% | |
| 52 [71] | Sep 2019 - May 2022<br>Pre-pandemic | Ampicillin: 74.5%<br>Cotrimoxazole: 58.82%<br>Ciprofloxacin: 49%<br>Levofloxacin: 37.25%<br>Cephalosporins: 33%<br>Amikacin/ tigecycline: 12.75%<br>Carbapenems: 11.76% | Overall MDR: 40.20%<br>E. coli MDR: 23.53%<br>Klebsiella spp. MDR: 48.15%, XDR: 22.22%, PDR: 3.70%<br>Enterococcus spp. MDR: 53.85%<br>Pseudomonas spp. MDR: 50.00%, XDR: 12.50%<br>Proteus spp. MDR: 100%, XDR: 50.00% |
| 53 [72] | Jan 2020 - Mar 2022<br>Pre-pandemic | NA | NA |
| 54 [73] | Mar 2017 - Feb 2018<br>Mar 2020 - Feb 2021<br>Pre-pandemic | NA | NA |
| 55 [74] | Feb 2019 - Ju 2020<br>Pre-pandemic | NR | NR |
| 56 [75] | 2017 - 2022<br>Pre-pandemic | NR | NR |
| 57 [76] | Jun 2018 - Jun 2020<br>Pre-pandemic | Total: Amp/Sul - 31.34%, Piper/Tazo - 17.39%, Ciprofloxacin - 20.00%, Levofloxacin - 17.31%, Cefepime - 20.92%, Ceftriaxone - 15.07%, Ceftazidime - 23.96%, Meropenem - 8.76%, Imipenem - 10.15%<br>E. coli: Amp/Sul - 58.06%, Piper/Tazo - 20.00%, Ciprofloxacin - 21.25%, Levofloxacin - 6.25%, Cefepime - 22.89%, Ceftriaxone - 17.50%, Ceftazidime - 26.51%, Meropenem - 0.00%, Imipenem - 1.18%<br>Klebsiella spp.: Amp/Sul - 20.00%, Piper/Tazo - 19.23%, Ciprofloxacin - 14.89%, Levofloxacin - 9.09%, Cefepime - 16.00%, Ceftriaxone - 14.29%, Ceftazidime - 20.41%, Meropenem - 10.00%, Imipenem - 10.00%<br>Pseudomonas spp.: Piper/Tazo - 28.57%, Ciprofloxacin - 18.18%, Levofloxacin - 11.11%, Cefepime - 23.81%, Ceftriaxone - 50.00%, Ceftazidime - 25.00%, Meropenem - 31.58%, Imipenem - 40.00%<br>Enterococcus spp.: Amp/Sul - 0.00%, Ciprofloxacin - 45.45%, Levofloxacin - 39.39% | NR |
| 58 [77] | Mar - Nov 2018<br>Mar - Nov 2019<br>Mar - Nov 2020<br>Pre-pandemic | NR | NR |
| 59 [78] | Mar 2020 - Nov 2022<br>pandemic | S. aureus: Oxacillin - 33.33%, Erythromycin - 55.56%, Clindamycin - 44.44%<br>E. faecalis: Gentamicin - 8.33% | MRSA: 33.33%<br>K. pneumoniae: ESBL: 63.16%, CRE: |

| | | | |
|---|---|---|---|
| | | E. faecium: Ampicillin - 8.33%, Gentamicin - 8.33%<br>E. coli: Ampicillin - 82.35%, Piper/Tazo - 5.88%, Ceftazidime - 17.65%, Ciprofloxacin - 23.53%, Cotrimoxazole - 58.82%<br>K. pneumoniae: Piper/Tazo - 84.21%, Ceftazidime - 84.21%, Meropenem - 63.16%, Amikacin - 78.95%, Ciprofloxacin - 78.95%, Colistin - 36.84%, Cotrimoxazole - 63.16%<br>A. baumannii: Meropenem - 100.00%, Amikacin - 78.57%, Ciprofloxacin - 100.00%, Cotrimoxazole - 92.86%<br>P. aeruginosa: Piper/Tazo - 14.29%, Ceftazidime - 14.29%, Meropenem - 14.29%, Amikacin - 7.14%, Ciprofloxacin - 14.29% | 63.16%, CTX-M + NDM: 26.32%, KPC: 36.84%<br>VRE: 0 |
| 60 [79] | 2005 - 2022<br>Pre-pandemic | NA | NR |
| 61 [80] | 2021<br>pandemic | Listeria monocytogenes: Clindamycin – 100% | NR |
| 62 [81] | Mar 2020 - Aug 2022<br>pandemic | Ampicillin - 92.05%<br>Amoxi/Clav - 85.23%<br>Cotrimoxazole - 85.23%<br>Nitrofurantoin - 70.45%<br>Ciprofloxacin - 75.00%<br>Levofloxacin - 73.86%<br>Cefuroxime - 77.27%<br>Ceftriaxone - 71.59%<br>Ceftazidime - 68.18%<br>Cefepime - 60.23%<br>Piper/Tazo - 21.59%<br>Imipenem - 14.77%<br>Meropenem - 9.09%<br>Gentamicin - 48.86% | Total MDR: 100% |
| 63 [82] | 1990 - 2021<br>Pre-pandemic | NA | NR |
| 64 [83] | Apr 2020 - Dec 2020<br>pandemic | NA | NA |
| 65 [84] | Jul 2021<br>pandemic | NA | NA |
| 66 [85] | Apr 2019 - Mar 2020<br>Jul 2021 - Jun 2022<br>Pre-pandemic | Prepandemic:<br>Acinetobacter spp.: Amoxi/Clav - 94.57%, Ceftazidime - 93.75%, Ceftriaxone - 97.73%, Cefotaxime - 95.78%, Cefazolin - 100%, Cefepime - 95.65%, Imipenem - 90.58%, Meropenem - 88.76%, Ciprofloxacin - 92.23%, Levofloxacin - 88.68%, Piper/Tazo - 86.76%, Colistin - 3.37%, Gentamicin - 86.42%, Aztreonam - 96.25% | prepandemic:<br>MDR Acinetobacter spp.: 84.40%<br>MDR E. coli: 20.99%<br>MDR Klebsiella spp.: 53.36%<br>MDR Proteus spp.: 74.19%<br>MDR Pseudomonas spp.: 57.03% |

E. coli: Amoxi/Clav - 29.52%, Ceftazidime - 31.68%, Ceftriaxone - 35.58%, Cefotaxime - 26.92%, Cefazolin - 57.14%, Cefepime - 33.33%, Imipenem - 16.49%, Meropenem - 2.60%, Ciprofloxacin - 39.82%, Levofloxacin - 52%, Piper/Tazo - 0%, Colistin - 4.69%, Gentamicin - 36.94%, Aztreonam - 27%
Klebsiella spp.: Amoxi/Clav - 66.36%, Ceftazidime - 65.31%, Ceftriaxone - 65.34%, Cefotaxime - 137%, Cefazolin - 77.16%, Cefepime - 61.68%, Imipenem - 40.60%, Meropenem - 45.68%, Ciprofloxacin - 59.76%, Levofloxacin - 55.56%, Piper/Tazo - 71.43%, Colistin - 0.38%, Gentamicin - 44.62%, Aztreonam - 56.35%
Proteus spp.: Amoxi/Clav - 78.13%, Ceftazidime - 72.95%, Ceftriaxone - 76.15%, Cefotaxime - 80.36%, Cefazolin - 94.12%, Cefepime - 54.80%, Imipenem - 46.67%, Meropenem - 19.79%, Ciprofloxacin - 68.33%, Levofloxacin - 86.96%, Piper/Tazo - 0%, Colistin - 100%, Gentamicin - 76.59%, Aztreonam - 27.20%
Pseudomonas spp.: Amoxi/Clav - 100%, Ceftazidime - 60.92%, Ceftriaxone - 82.35%, Cefotaxime - 75%, Cefazolin - 100%, Cefepime - 76.47%, Imipenem - 58.44%, Meropenem - 65%, Ciprofloxacin - 55.68%, Levofloxacin - 60.78%, Piper/Tazo - 36.84%, Colistin - 0%, Gentamicin - 61.29%, Aztreonam - 50%
S. aureus: Ciprofloxacin - 61.67%, Clindamycin - 76.25%, Clarithromycin - 56.74%, Doxycycline - 40.08%, Erythromycin - 74.17%, Linezolid - 0.22%, Penicillin - 98.34%, Rifampicin - 53.16%, Tetracycline - 62.57%, Oxacillin - 72.99%, Vancomycin - 16.67%, Teicoplanin - 26.31%
CoNS: Ciprofloxacin - 80.87%, Clindamycin - 66.93%, Clarithromycin - 67.60%, Doxycycline - 54.74%, Erythromycin - 80.65%, Linezolid - 23.88%, Penicillin - 93.33%, Rifampicin - 45.24%, Tetracycline - 71.43%, Oxacillin - 81.89%, Vancomycin - 15.79%, Teicoplanin - 26.67%
Streptococcus spp.: Ciprofloxacin - 100%, Clindamycin - 29.31%, Clarithromycin - 49.12%, Doxycycline - 10.57%, Erythromycin - 52.55%, Linezolid - 0%, Penicillin - 85.11%, Rifampicin - 20%, Tetracycline - 35.04%, Oxacillin - 91.89%, Vancomycin - 1.11%
Enterococcus spp.: Ciprofloxacin - 86.05%, Clindamycin - 100%, Clarithromycin - 100%, Doxycycline - 80.65%, Erythromycin - 100%, Linezolid - 4.54%, Penicillin - 38.09%, Tetracycline - 75%, Vancomycin - 29.17%, Teicoplanin - 29.27%

Pandemic:
Acinetobacter spp.: Amoxi/Clav - 100%, Ceftazidime - 96.01%, Ceftriaxone - 97.12%, Cefotaxime - 95.43%, Cefazolin - 100%, Cefepime - 84.43%, Imipenem - 90.17%, Meropenem - 88.48%, Ciprofloxacin - 94.83%, Levofloxacin - 88.88%, Piper/Tazo - 91.62%, Colistin - 18.09%, Gentamicin - 85.19%, Aztreonam - 81.03%

MRSA: 9.13%
MDR CoNS: 68.75%
MDR Enterococcus: 44.44%
MDR Streptococcus spp.: 14.59%
MDR Other: 43.14%

pandemic:
MDR Acinetobacter spp.: 66.43%, PDR: 4.59%
MDR E. coli: 16.07%
MDR Klebsiella spp.: 49.39%
MDR Proteus spp.: 35.51%, PDR: 7.25%
MDR Pseudomonas spp.: 29.32%
MRSA: 6.36%
MDR CoNS: 16.02%
MDR Enterococcus: 8.29%
MDR Streptococcus spp.: 1.04%
MDR Other: 39.17%, PDR: 1.38%

| | | | |
|---|---|---|---|
| 67 [86] | 2016 - 2020<br>Pre-pandemic | E. coli: Amoxi/Clav - 36.58%, Ceftazidime - 38.55%, Ceftriaxone - 33.95%, Cefotaxime - 33.78%, Cefazolin - 51.24%, Cefepime - 25.97%, Imipenem - 6.14%, Meropenem - 9.57%, Ciprofloxacin - 43.54%, Levofloxacin - 28.76%, Piper/Tazo - 24%, Colistin - 32.46%, Gentamicin - 37.73%, Aztreonam - 27.43%<br>Klebsiella spp.: Amoxi/Clav - 62.98%, Ceftazidime - 69.68%, Ceftriaxone - 67.30%, Cefotaxime - 65.55%, Cefazolin - 79.32%, Cefepime - 55.30%, Imipenem - 45.56%, Meropenem - 44.35%, Ciprofloxacin - 61.30%, Levofloxacin - 59%, Piper/Tazo - 61.34%, Colistin - 20.51%, Gentamicin - 64.85%, Aztreonam - 73.60%<br>S. aureus: Oxacillin - 35.70%, Clindamycin - 52.10%, Ciprofloxacin - 27.40%, Erythromycin - 67.10%, Gentamicin - 22.40%, Linezolid - 0.30%, Teicoplanin - 12.20%, Vancomycin - 14.30%, Cotrimoxazole - 14.00%<br>MRSA: Clindamycin - 78.90%, Ciprofloxacin - 53.10%, Erythromycin - 94.30%, Gentamicin - 44.00%, Linezolid - 0.40%, Teicoplanin - 13.60%, Vancomycin - 0.00%, Cotrimoxazole - 21.70%<br>Enterococcus spp.: Ampicillin - 12.15%, Ciprofloxacin - 50.13%, Linezolid - 0.00%, Vancomycin - 2.23%<br>A. baumannii: Ceftazidime - 72.70%, Cefepime - 61.50%, Piper/Tazo - 33.33%, Meropenem - 42.90%, Ciprofloxacin - 52.60%, Gentamicin - 44.40%<br>P. aeruginosa: Ceftazidime - 33.50%, Cefepime - 39.70%, Piper/Tazo - 26.30%, Meropenem - 44.80%, Ciprofloxacin - 35.20%, Gentamicin - 38.40%<br>K. pneumoniae: Amoxi/Clav - 49.60%, Cefuroxime - 36.30%, Cefotaxime - 33.90%, Ceftazidime - 77.80%, Cefepime - 61.30%, Piper/Tazo - 27.50%, Ertapenem - 26.70%, Meropenem - 26.60%, Ciprofloxacin - 37.00%, Gentamicin - 25.90%, Cotrimoxazole - 39.60%<br>E. coli: Amoxi/Clav - 39.80%, Cefuroxime - 14.80%, Cefotaxime - 12.70%, Cefepime - 22.20%, Piper/Tazo - 8.40%, Ertapenem - 4.30%, Meropenem - 4.00%, Ciprofloxacin - 24.70%, Gentamicin - 8.50%, Cotrimoxazole - 35.70%<br>Proteus spp.: Amoxi/Clav - 43.00%, Cefuroxime - 31.60%, Cefotaxime - 17.70%, Cefepime - 33.70%, Piper/Tazo - 6.20%, Ertapenem - 2.00%, Meropenem - 0.00%, Ciprofloxacin - 26.20%, Gentamicin - 31.50%, Cotrimoxazole - 55.70%<br>Enterobacter spp.: Amoxi/Clav - 84.30%, Cefuroxime - 65.10%, Cefotaxime - 30.00%, Cefepime - 18.20%, Piper/Tazo - 13.00%, Ertapenem - 20.00%, Meropenem - 26.70%, Ciprofloxacin - 17.80%, Gentamicin - 11.50%, Cotrimoxazole - 30.00%<br>Salmonella spp.: Amoxi/Clav - 25.00%, Ertapenem - 0.00%, Meropenem - 0.00%, Ciprofloxacin - 22.80%, Gentamicin - 22.20%, Cotrimoxazole - 5.00%<br>Other Enterobacteriaceae: Amoxi/Clav - 85.10%, Cefuroxime - 56.50%, Cefotaxime - 36.70%, Cefepime - 50.00%, Piper/Tazo - 10.70%, Ertapenem - | Total period:<br>MDR S. aureus: 41.77%, MRSA: 37.81%<br>VRE: 2.03%<br>MDR K. pneumoniae: 24.37%, ESBL: CD, CRE: 7.54%<br>MDR A. baumanii: 35.71%<br>MDR P. aeruginosa: 29.23%<br>MDR E. coli: 9.79%, ESBL: 7.65%, CRE: 4.02%<br>MDR Proteus spp.: 15.97%<br>MDR Enterobacter spp.: 15.00%<br>MDR Salmonella spp.: 4.11%<br>MDR Other: 45.16% |

| | | | |
|---|---|---|---|
| 68 [87] | Jan 2017 - Dec 2020<br>Pre-pandemic | 12.50%, Meropenem - 26.70%, Ciprofloxacin - 27.50%, Gentamicin - 24.40%, Cotrimoxazole - 38.00%<br>K. pneumoniae: Ampicillin - 94.29%, Amoxi/Clav - 78.57%, Amikacin - 40.00%, Aztreonam - 77.14%, Cefepime - 72.86%, Cefotaxime - 81.43%, Cefoxitin - 67.14%, Ceftazidime - 82.86%, Ceftriaxone - 81.43%, Cefuroxime - 15.71%, Ciprofloxacin - 82.86%, Levofloxacin - 70.00%, Moxifloxacin - 62.86%, Colistin - 11.43%, Ertapenem - 74.29%, Imipenem - 58.57%, Meropenem - 71.43%, Gentamicin - 51.43%, Piper/Tazo - 77.14%, Tobramycin - 77.14%, Tetracycline - 57.14%, Tigecycline - 14.29%, Chloramphenicol - 62.86%<br>E. Coli: Ampicillin - 66.67%, Amoxi/Clav - 18.52%, Amikacin - 3.70%, Aztreonam - 14.81%, Cefepime - 11.11%, Cefotaxime - 22.22%, Cefoxitin - 7.41%, Ceftazidime - 29.63%, Ceftriaxone - 33.33%, Cefuroxime - 11.11%, Ciprofloxacin - 29.63%, Levofloxacin - 37.04%, Moxifloxacin - 25.93%, Colistin - 18.52%, Ertapenem - 3.70%, Imipenem - 3.70%, Meropenem - 3.70%, Gentamicin - 11.11%, Piper/Tazo - 11.11%, Tobramycin - 14.81%, Tetracycline - 29.63%, Tigecycline - 3.70%, Chloramphenicol - 11.11%<br>P. mirabillis: Ampicillin - 50.00%, Amoxi/Clav - 50.00%, Amikacin - 12.50%, Aztreonam - 0.00%, Cefepime - 12.50%, Cefotaxime - 12.50%, Cefoxitin - 12.50%, Ceftazidime - 25.00%, Ceftriaxone - 25.00%, Cefuroxime - 12.50%, Ciprofloxacin - 37.50%, Levofloxacin - 37.50%, Moxifloxacin - 25.00%, Colistin - 100.00%, Ertapenem - 0.00%, Imipenem - 75.00%, Meropenem - 0.00%, Gentamicin - 25.00%, Piper/Tazo - 12.50%, Tobramycin - 37.50%, Tetracycline - 100.00%, Tigecycline - 62.50%, Chloramphenicol - 50.00%<br>A. baumanii: Amoxi/Clav - 26.67%, Aztreonam - 37.78%, Cefepime - 88.89%, Cefotaxime - 88.89%, Ceftazidime - 88.89%, Ceftriaxone - 88.89%, Cefuroxime - 88.89%, Ciprofloxacin - 95.56%, Levofloxacin - 80.00%, Colistin - 8.89%, Imipenem - 84.44%, Meropenem - 95.56%, Gentamicin - 97.78%, Piper/Tazo - 93.33%, Tetracycline - 73.33%, Tigecycline - 11.11%, Tobramycin - 71.11%<br>P. aeruginosa: Cefepime - 43.33%, Cefotaxime - 50.00%, Ceftazidime - 50.00%, Ciprofloxacin - 50.00%, Levofloxacin - 46.67%, Colistin - 6.67%, Ertapenem - 6.67%, Imipenem - 43.33%, Meropenem - 50.00%, Gentamicin - 43.33%, Piper/Tazo - 50.00%, Tetracycline - 6.67%, Tigecycline - 40.00%, Tobramycin - 50.00%<br>Other NFB: Amoxi/Clav - 18.18%, Aztreonam - 81.82%, Cefepime - 72.73%, Cefotaxime - 54.55%, Ceftazidime - 45.45%, Ceftriaxone - 63.64%, Cefuroxime - 18.18%, Ciprofloxacin - 81.82%, Levofloxacin - 45.45%, Colistin - 36.36%, Ertapenem - 9.09%, Imipenem - 45.45%, Meropenem - 45.45%, Gentamicin - 63.64%, Piper/Tazo - 45.45%, Tetracycline - 63.64%, Tigecycline - 45.45%, Tobramycin - 81.82%<br>MRSA: Ciprofloxacin - 67.74%, Clindamycin - 74.19%, Erythromycin - 82.26%, | pre pandemic:<br>MRSA: 96.08%<br>MDR CoNS: 46.45%<br>MDR E. faecalis: 61.11%<br>MDR A. baumanii: 96.77%, PDR: 3.23%<br>MDR E. coli: 13.33%<br>MDR P. aeruginosa: 66.67%, PDR: 6.67%<br>MDR K. pneumoniae: 50.00%<br><br>pandemic:<br>MRSA: 81.82%<br>MDR CoNS: 28.89%<br>MDR E. faecalis: 37.50%<br>MDR A. baumanii: 100%<br>MDR P. aeruginosa: 20.00%<br>MDR K. pneumoniae: 50.00% |

| | | | |
|---|---|---|---|
| | | Gentamicin - 56.45%, Levofloxacin - 24.19%, Linezolid - 3.23%, Moxifloxacin - 64.52%, Oxacillin - 93.55%, Penicillin - 93.55%, Rifampicin - 48.39%, Teicoplanin - 12.90%, Tetracycline - 59.68%, Vancomycin - 6.45%<br>CoNS: Ciprofloxacin - 59.43%, Clindamycin - 59.12%, Erythromycin - 72.96%, Gentamicin - 49.69%, Levofloxacin - 48.11%, Linezolid - 10.06%, Moxifloxacin - 56.92%, Oxacillin - 73.27%, Penicillin - 78.93%, Rifampicin - 44.97%, Teicoplanin - 8.81%, Tetracycline - 69.81%, Tigecycline - 0.31%, Vancomycin - 6.60%<br>E. faecalis: Ciprofloxacin - 83.33%, Clindamycin - 19.05%, Erythromycin - 88.10%, Gentamicin - 11.90%, Levofloxacin - 52.38%, Linezolid - 11.90%, Moxifloxacin - 14.29%, Penicillin - 30.95%, Teicoplanin - 9.52%, Tetracycline - 59.52%, Tigecycline - 2.38%, Vancomycin - 21.43% | |
| 69 [88] | Jan - Jun 2020 pandemic | C. difficile:<br>Vancomycin: 68.00%<br>Metronidazole: 50.00% | NA |
| 70 [89] | Sep 2017 - Oct 2021 Pre-pandemic | NA | Total CRE E. Coli + Klebsiella spp.: 100% |
| 71 [90] | Oct 2019 - Oct 2020 Pre-pandemic | K. pneumoniae: Ciprofloxacin - 100%, Levofloxacin - 100%, Cephalosporins - 100%, Carbapenems - 100%, Piper/Tazo - 100%, Amikacin - 29.4%, Gentamicin - 45.30%, Ceftazidime/Avibactam - 17.60%, Cotrimoxazole - 74.50%<br>Acinetobacter spp.: Ciprofloxacin - 100%, Levofloxacin - 100%, Cephalosporins - 100%, Carbapenems - 100%, Piper/Tazo - 100%, Amikacin - 100%, Gentamicin - 100%, Cotrimoxazole - 54.50%, Ampi/Sulb - 18.20%, Minocycline - 18.20%<br>P. aeruginosa: Ciprofloxacin - 100%, Levofloxacin - 100%, Cephalosporins - 100%, Carbapenems - 100%, Piper/Tazo - 30%, Amikacin - 100%, Gentamicin - 100%, Ceftazidime/Avibactam - 0% | Total MDR: 100%<br>K. pneumoniae: ; ESBL: 80.39%, CRE: 80.39%; KPC: 33.33%, TEM: 37.25%, NDM: 17.65%, OXA: 29.41%, SHV: 43.14%<br>P. Aeruginosa: CRO: 20.00%; IMP: 10.00%, VIM: 20.00%<br>Acinetobacter spp.: CRO: 81.82%; VIM: 9.09%, OXA: 90.90%, |
| 72 [91] | Jan 2022 - Aug 2023 Post-pandemic | S. aureus: Ampicillin - 84.20%, Amoxicillin - 53.84%, Clarythromycin - 47.80%, Cefuroxime - 17.39%, Ciprofloxacin - 7.81%, Erythromycin - 47.22%, Gentamicin - 14.92%, Levofloxacin - 5.71%, Ofloxacin - 20.00%, Tetracycline - 43.47%, Tobramycin - 12.12%<br>CoNS: Ampicillin - 87.50%, Amoxicillin - 40.00%, Clarythromycin - 54.54%, Cefuroxime - 15.38%, Ciprofloxacin - 12.50%, Erythromycin - 60.86%, Gentamicin - 26.67%, Levofloxacin - 14.20%, Tetracycline - 52.94%, Tobramycin - 7.69%<br>Group A Strep.: Tetracycline - 50.00%<br>Enterococcus spp.: Ampicillin - 66.67%, Tetracycline - 50.00%, Vancomycin - 25.00%<br>Klebsiella spp.: Ampicillin - 100.00%, Cefuroxime - 66.67%<br>Proteus spp.: Ampicillin - 66.67%, Amoxicillin - 100.00%, Cefuroxime - 50.00%<br>P. aeruginosa: Ampicillin - 100.00% | MRSA: 5.26%<br>VRE: 25.00% |

| | | | |
|---|---|---|---|
| | | E. coli: Ampicillin - 100.00%, Amoxicillin - 50.00%<br>Enterobacter spp.: Ampicillin - 100.00%<br>Haemophilus spp.: Ampicillin - 50.00%, Amoxicillin - 100.00%<br>S. marcescens: Cefuroxime - 100.00% | |
| 73 [92] | Mar 2020 - Jan 2021 pandemic | NA | NA |
| 74 [93] | Jan 2020 - Dec 2021 pandemic | E. coli: Amikacin - 14.29%, Ampicillin - 100%, Aztreonam - 71.43%, Cephazolin - 100%, Cefepime - 85.71%, Ceftazidime - 100%, Ceftriaxone - 100%, Cefuroxime - 100%, Cefotaxime - 100%, Ciprofloxacin - 42.86%, Tetracycline - 57.14%, Gentamicin - 57.14%, Moxifloxacin - 50%, Norfloxacin - 42.86%, Piper/Tazo - 28.57%, Cotrimoxazole - 28.57%, Tigecycline - 14.29%, Tobramycin - 57.14%, Imipenem - 28.57%, Meropenem - 14.29%, Ertapenem - 28.57%<br>K. pneumoniae: Amikacin - 12.50%, Ampicillin - 100%, Aztreonam - 62.50%, Cephazolin - 100%, Cefepime - 81.25%, Ceftazidime - 100%, Ceftriaxone - 81.25%, Cefuroxime - 100%, Cefotaxime - 100%, Ciprofloxacin - 68.75%, Tetracycline - 43.75%, Gentamicin - 42.75%, Moxifloxacin - 37.50%, Norfloxacin - 62.50%, Piper/Tazo - 50%, Cotrimoxazole - 75%, Tigecycline - 37.50%, Tobramycin - 68.75%, Imipenem - 62.50%, Meropenem - 43.75%, Ertapenem - 50% | Total ESBL: 100%<br>E. coli CTX: 92.86%, SHV: 7.14%, TEM: 78.57%, tet(A): 42.86%, tet(B): 7.14%<br>K. pneumoniae CTX: 71.88%, SHV: 100%, TEM: 43.75%, tet(A): 3.13% |
| 75 [94] | Jan 2016 - Jun 2022 Pre-pandemic | E. coli: Carbapenems - 18.75%, Piper/Tazo - 31.25%, Ceftazidime - 50%, Levofloxacin - 56.25%<br>Klebsiella spp.: Carbapenems - 16.67%, Piper/Tazo - 41.67%, Ceftazidime - 50%, Levofloxacin - 58.34%, Ceftriaxone - 58.34%<br>Others: NR | NR |
| 76 [95] | Jan 2016 -Dec 2021 Pre-pandemic | NR | MDR S. aureus: 27.27%<br>MDR Enterococcus: 30.30%<br>MDR CoNS: 27.78%<br>MDR viridans strep.: 40.00%<br>MDR group D strep.: 33.33%<br>MDR Streptococcus spp.: 35.29%<br>MDR P. aeruginosa: 44.44%<br>MDR H. influenzae: 33.33%<br>MDR E. coli: 25.00%<br>MDR Salmonella: 33.33%<br>MDR Other: 100% |
| 77 [96] | Jan 2021 - Jun 2021 pandemic | E. coli: Aminoglycosides - 1.80%, Quinolones - 17.80%, Cotrimoxazole - 25.20%, Tetracycline - 1.80%<br>Klebsiella spp.: Aminoglycosides - 4.84%, Quinolones - 15.32%, Cotrimoxazole - 14.51%, Tetracycline - 2.50%<br>Pseudomonas spp.: Aminoglycosides - 11.29%, Quinolones - 17.74% | E. coli: MDR: 9.13%, ESBL: 6.39%, CRE: 0.46%<br>Klebsiella spp.: MDR: 12.10%, XDR: 2.42%, ESBL: 11.29%, CRE: 7.26%<br>Pseudomonas spp.: MDR: 24.19%, XDR: |

| | | | |
|---|---|---|---|
| | | Acinetobacter spp.: Aminoglycosides - 52.94%, Quinolones - 47.05%,<br>Cotrimoxazole - 52.94%<br>Others: NR | 6.45%, CRO: 19.35%<br>Acinetobacter spp.: MDR: 58.82%, XDR: 23.53%,CRO: 52.54%<br>Other: NR |
| 78 [97] | Jan 2019 - Jun 2022<br>Pre-pandemic | Prepandemic:<br>Cotrimoxazole: 76.09%<br>Cefepime: 100.00%<br>Cefoxitine: 100.00%<br>Gentamicin: 82.61%<br>Tobramycin: 100.00%<br>Amikacin: 82.61%<br>Ciprofloxacin: 97.83%<br>Ertapenem: 100.00%<br>Imipenem: 86.05%<br>Meropenem: 88.37%<br>Piper/Tazo: 100.00%<br>Ceftazidime/Avibactam: 53.85%<br>Colistin: 34.88%<br><br>Pandemic:<br>Nitrofurantoin: 93.33%<br>Fosfomycin: 36.00%<br>Cotrimoxazole: 82.93%<br>Cefepime: 95.12%<br>Cefoxitine: 93.94%<br>Gentamicin: 52.50%<br>Tobramycin: 94.74%<br>Amikacin: 77.50%<br>Ciprofloxacin: 97.56%<br>Ertapenem: 100.00%<br>Imipenem: 80.49%<br>Meropenem: 92.50%<br>Piper/Tazo: 97.50%<br>Ceftazidime/Avibactam: 75.86%<br>Colistin: 46.34% | Unspecified Prepandemic:<br>CRE: 82.61%, NDM: 34.78%, OXA: 21.74%, KPC: 19.57%, VIM: 2.17%, mcr-1: 6.52%<br><br>Unspecified Pandemic:<br>CRE: 82.93%, NDM: 21.95%, OXA: 39.02%, KPC: 9.76%, VIM: 2.44%, mcr-1: 4.88% |
| 79 [98] | 2019 - 2020<br>Pre-pandemic | NA | NA |
| 80 [99] | Mar - Dec 2020<br>pandemic | NR | MDR E. coli: 100% |

| | | | |
|---|---|---|---|
| 81 [100] | Jan 2019 - Feb 2020<br>Mar 2020 - May 2021<br>Pre-pandemic | NR | NR |
| 82 [101] | Jul 2020 - Jun 2022<br>pandemic | TB Rifampicin: 2.22% | MDR TB: 2.22% |
| 83 [102] | Jan 2020 - Jan 2022<br>pandemic | Cephalosporins: 12.27%<br>Macrolides: 11.04%<br>Penicillin: 22.09%<br>Aminoglycosides: 9.41%<br>Tetracycline: 8.18%<br>Quinolones: 6.75%<br>Carbapenems: 4.29%<br>Glycopeptides: 6.13%<br>Nitroimidazoles: 3.68%<br>Other: 3.27% | Unspecified MDR: 30.88% |
| 84 [103] | Jul 2021<br>pandemic | NA | NR |
| 85 [104] | 2020 - 2021<br>pandemic | NR | NR |
| 86 [105] | Jan 2022 - Sept 2023<br>Post-pandemic | NR | NR |
| 87 [106] | Sep - Dec 2018<br>Sep - Dec 2020<br>Sep - Dec 2022<br>Pre-pandemic | Pre-pandemic<br>E. coli: Amikacin - 3.55%, Amoxi/Clav - 21.34%, Ceftazidime - 7.11%, Levofloxacin - 28.45%, Meropenem - 0.39%, Nitrofurantoin - 6.71%<br>Klebsiella spp.: Amikacin - 8.97%, Amoxi/Clav - 34.61%, Ceftazidime - 20.51%, Imipenem - 6.41%, Levofloxacin - 14.1%, Meropenem - 6.41%<br>Pseudomonas spp.: Amikacin - 14.28%, Ceftazidime - 21.42%, Imipenem - 14.28%, Levofloxacin - 21.42%, Meropenem - 14.28%<br>Proteus spp.: Amikacin - 11.76%, Amoxi/Clav - 32.35%, Ceftazidime - 14.7%, Levofloxacin - 32.35%, Meropenem - 2.94%<br>Enterococcus spp.: Ampicillin - 20.23%, Fosfomycin - 1.19%, Levofloxacin - 38.09%, Nitrofurantoin - 3.57%, Penicillin - 34.52%<br>Staphylococcus spp.: Cotrimoxazole - 26.66%, Levofloxacin - 13.33%, Linezolid - 13.33%, Penicillin - 46.66%<br><br>Pandemic:<br>E. coli: Amikacin - 13.61%, Amoxi/Clav - 32.78%, Ceftazidime - 13.06%, Fosfomycin - 0.28%, Imipenem - 0.83%, Levofloxacin - 32.22%, Nitrofurantoin - 2.50% | |
